# Reperfusion After Fibrinolytic Therapy (RAFT): An open-label, multi-centre, randomised controlled trial of bivalirudin versus heparin in rescue percutaneous coronary intervention

**DOI:** 10.1371/journal.pone.0259148

**Published:** 2021-10-26

**Authors:** Amir Faour, Nicholas Collins, Trent Williams, Arshad Khan, Craig P. Juergens, Sidney Lo, Darren L. Walters, Derek P. Chew, John K. French

**Affiliations:** 1 Department of Cardiology, Liverpool Hospital, Sydney, New South Wales, Australia; 2 University of New South Wales, Sydney, New South Wales, Australia; 3 Department of Cardiology, John Hunter Hospital, Newcastle, New South Wales, Australia; 4 University of Queensland, Brisbane, Queensland, Australia; 5 Department of Cardiology, The Prince Charles Hospital, Brisbane, Queensland, Australia; 6 Department of Cardiology, Flinders Medical Centre, Adelaide, South Australia, Australia; 7 Ingham Institute and Western Sydney University, Sydney, New South Wales, Australia; Federico II University, ITALY

## Abstract

**Background:**

The safety and efficacy profile of bivalirudin has not been examined in a randomised controlled trial of patients undergoing rescue PCI.

**Objectives:**

We conducted an open-label, multi-centre, randomised controlled trial to compare bivalirudin with heparin ± glycoprotein IIb/IIIa inhibitors (GPIs) in patients undergoing rescue PCI.

**Methods:**

Between 2010–2015, we randomly assigned 83 patients undergoing rescue PCI to bivalirudin (n = 42) or heparin ± GPIs (n = 41). The primary safety endpoint was any ACUITY (Acute Catheterization and Urgent Intervention Triage Strategy) bleeding at 90 days. The primary efficacy endpoint was infarct size measured by peak troponin levels as a multiple of the local upper reference limit (Tn/URL). Secondary endpoints included periprocedural change in haemoglobin adjusted for red cells transfused, TIMI (Thrombolysis in Myocardial Infarction) bleeding, ST-segment recovery and infarct size determined by the Selvester QRS score.

**Results:**

The trial was terminated due to slow recruitment and futility after an interim analysis of 83 patients. The primary safety endpoint occurred in 6 (14%) patients in the bivalirudin group (4.8% GPIs) and 3 (7.3%) in the heparin ± GPIs group (54% GPIs) (risk ratio, 1.95, 95% confidence interval [CI], 0.52–7.3, P = 0.48). Infarct size was similar between the two groups (mean Tn/URL, 730 [±675] for bivalirudin, versus 984 [±1585] for heparin ± GPIs, difference, 254, 95% CI, -283-794, P = 0.86). There was a smaller decrease in the periprocedural haemoglobin level with bivalirudin than heparin ± GPIs (-7.5% [±15] versus -14% [±17], difference, -6.5%, 95% CI, -0.83–14, P = 0.0067). The rate of complete (≥70%) ST-segment recovery post-PCI was higher in patients randomised to heparin ± GPIs compared with bivalirudin.

**Conclusions:**

Whether bivalirudin compared with heparin ± GPI reduces bleeding in rescue PCI could not be determined. Slow recruitment and futility in the context of lower-than-expected bleeding event rates led to the termination of this trial (ANZCTR.org.au, ACTRN12610000152022).

## Introduction

Patients undergoing rescue percutaneous coronary intervention (PCI) are at high risk of bleeding complications [1–4]. Bivalirudin is a direct thrombin inhibitor with pharmacokinetic and pharmacodynamic advantages over heparin. In studies over two decades ago, when bivalirudin was compared to heparin as an adjunctive antithrombin agent to fibrinolytic therapy, improved ischaemic endpoints and reduced bleeding has been reported [5–8]. Since then, conflicting safety and efficacy outcomes for bivalirudin relative to heparin have been reported in contemporary trials of patients undergoing primary PCI [9]. Several study-level meta-analyses report reduced major bleeding and variable mortality benefits with bivalirudin compared to heparin ± glycoprotein IIb/IIIa inhibitors, at the expense of an increased risk of acute stent thrombosis [10–12]. However, the majority of recent large-scale trials excluded fibrinolytic-treated patients [13–19], except for MATRIX (Minimising Adverse Haemorrhagic Events by Transradial Access Site and Systemic Implementation of Angiox), which included a small group of patients undergoing pharmaco-invasive PCI [20, 21].

As the safety and efficacy profile of bivalirudin has not been examined in patients undergoing rescue PCI, we conducted a randomised controlled trial comparing bivalirudin with heparin ± glycoprotein IIb/IIIa inhibitors in patients undergoing rescue PCI. We hypothesised that bivalirudin compared with heparin ± glycoprotein IIb/IIIa inhibitors will result in lower ACUITY (Acute Catheterization and Urgent Intervention Triage Strategy) [13] bleeding at 90 days and would be similarly efficacious as assessed by infarct size determined by biomarker levels.

## Methods

### Trial design

The Reperfusion After Fibrinolytic Therapy (RAFT) trial was an investigator-initiated, open-label, multi-centre, randomised clinical trial that compared bivalirudin with heparin ± glycoprotein IIb/IIIa inhibitors in patients with STEMI undergoing rescue PCI (principal investigator JKF) (S1 Fig). The Medicines Company funded the trial by providing a research grant-in-aid and the study drug but had no access to the data and no role in the design, conduct, analysis or reporting of the trial. Project management occurred at Flinders Co-ordinating Centre and The South Australian Health and Medical Research Institute in Adelaide, South Australia. The trial protocol received multi-site approval from the Concord Repatriation General Hospital Human Research Ethics Committee, Sydney, Australia. The trial was performed in accordance with the ethical standards of the Helsinki Declaration of Good Clinical Practice.

### Patients

Patients ≥ 18 years of age presenting with STEMI < 12 hours after symptom onset, who failed to achieve 50% ST-segment recovery at 60–90 minutes post-fibrinolysis, and who subsequently required rescue PCI were eligible for enrolment. Patients with STEMI required chest pain of ≥ 30 minutes and ST-segment elevation ≥ 1 mm in 2 contiguous leads (or ≥ 2 mm in V2-V3) or new left bundle branch block, together with elevated levels of cardiac biomarkers (troponin I or T > upper reference limit). Exclusion criteria were known hypersensitivity or contraindication to study medications, hypertension with blood pressure persistently > 180/110 mm Hg, significant bleeding disorder or recent major bleeding within 3 months, warfarin therapy, known history of intracranial haemorrhage or trauma, known history of ischaemic stroke or recurrent transient ischaemic attacks, severe renal impairment (creatinine clearance < 30 ml/min or creatine ≥ 250 μmol/L), unavailability for follow up and female patients of childbearing potential.

Witnessed verbal consent was obtained after the decision to perform rescue PCI and before randomisation. Patients confirmed further participation by providing written informed consent within the following 24 hours. In August 2014, to increase recruitment, the steering committee amended the protocol to include all patients undergoing pharmaco-invasive PCI <24 hours post-fibrinolysis. However, no patients were recruited following this amendment as the trial was terminated due to slow recruitment. We kept a screening log of potentially eligible patients not recruited from the 2 highest recruiting centres.

### Study protocol and randomisation

In a 1:1 ratio and an open-label fashion, patients were randomly assigned to receive either a bolus and infusion of bivalirudin or a bolus of intravenous heparin ± glycoprotein IIb/IIIa inhibitors. Randomisation occurred after the decision to perform rescue PCI. Patients who received glycoprotein IIb/IIIa inhibitors before PCI were eligible if it was considered safe to stop the infusion prior to randomisation. We randomised patients using a web-based system in randomly permuted blocks, using computer-generated sequences and central concealment.

Unless contraindicated, aspirin (300 mg) was given immediately at presentation or pre-PCI and continued indefinitely thereafter at 100–150 mg/day. Clopidogrel loading dose of 300 mg was recommended and continued at 75 mg/day post-PCI and was advised for at least 12 months. Fibrinolytic therapy consisted of weight-adjusted tenecteplase to a maximum of 50 mg (30 mg for < 60 kg, 35 mg for 60–69 kg, 40 mg for 70–79 kg, 45 mg for 80–89 kg and 50 mg for > 90 kg). Adjunctive antithrombin therapies were unfractionated heparin (60 units/kg bolus, maximum 4000 units + 12 units/kg/hr infusion, max 1000 units/hr, until angiography), or enoxaparin (30 mg intravenous bolus + 1 mg/kg subcutaneously, maximum 100 mg, administered within 15 minutes of the bolus; for patients ≥75 years old, the bolus was omitted and 0.75 mg/kg was administered subcutaneously). Bivalirudin was administered intravenously as a bolus and infusion (0.75 mg/kg bolus + 1.75 mg/kg/hr infusion) as soon as PCI was planned and continued for 4 hours post-PCI. The use of ‘bailout’ glycoprotein IIb/IIIa inhibitors was allowed in the bivalirudin arm if required (recurrent thrombus formation or major side branch occlusion endangering the procedural success and clinical outcome). Heparin was given immediately before PCI at a dose of 70–100 units per kilogram, and this was adjusted to 50–70 units per kilogram in patients receiving glycoprotein IIb/IIIa inhibitors. A target activated clotting time of 250 to 300 seconds during PCI was recommended with heparin monotherapy and 200 to 250 s with heparin plus glycoprotein IIb/IIIa inhibitors. Guideline-based medical therapies were recommended unless contraindicated. The trial protocol is available from the Supporting Information section of the article (S2 File).

### Endpoints

Follow up occurred at 30 and 90 days. Clinical events were site reported and were not adjudicated by a committee due to funding limitations. The primary safety endpoint was any ACUITY bleeding (major and minor) at 90 days. The primary efficacy endpoint pre-specified in the protocol was the area under the curve of creatine kinase-MB assayed in a core laboratory. Due to trial termination, funds did not permit core laboratory measurement of creatine kinase-MB, which was no longer assayed routinely. As we reported during trial recruitment the strong correlation between peak troponin T levels and cardiac magnetic resonance imaging derived infarct size [22], and a close correlation between troponin T and creatine kinase-MB levels [23], we used the peak troponin I or T level as the primary efficacy endpoint. Troponin levels were collected at baseline, 60–90 minutes post fibrinolysis, pre-PCI and every 6–8 hours for the first 24 hours and then daily until the highest troponin level was detected. Peak troponin I or T levels were divided by the upper reference limit of the corresponding assay to correct for the different troponin I assays used at each site.

Secondary outcomes included periprocedural changes in haemoglobin level, TIMI (Thrombolysis in Myocardial Infarction) bleeding (major, minor and minimal) [24], infarct size determined by the Selvester QRS score [25] and post-PCI ST-segment recovery in the lead with maximum ST-segment elevation [26, 27]. Periprocedural change in haemoglobin level was expressed as the per cent change between the level before coronary angiography and the next day with adjustment for units of red blood cells transfused (1 unit of blood = 10 g/L of circulating haemoglobin [28]).

ECG analysis was performed by the first author (AF) using digital callipers blinded to treatment allocation, the timing of ECGs and clinical outcomes. Infarct size estimation by the Selvester QRS score was performed on pre-discharge ECGs using the 32-point system (each point represents ~3% of the left ventricular myocardium). QRS scores were adjusted for bundle branch block and left ventricular hypertrophy. Per protocol, ECGs were acquired before fibrinolysis, post-fibrinolysis (60–90 minutes) and post-PCI (<24 hours). ST-segment recovery was expressed as per cent change from baseline in the lead with maximum ST-segment elevation. With the TP-segment as the iso-electric line, ST-segment elevation was measured to the nearest 0.05 mV at 20 milliseconds after the J-point. For statistical analysis, the per cent change in ST-segment recovery was classified as complete (>70%), partial (30% to 70%), and no recovery (<30%) [29]. ECGs were deemed unsuitable if they were of poor quality or with conduction abnormalities such as left bundle branch block or paced rhythm.

Other clinical endpoints included TIMI flow grade, death, recurrent myocardial infarction, stent thrombosis, target vessel revascularisation, stroke, and hospitalisation for heart failure at 30 and 90 days. TIMI flow grade was to be assessed in a core laboratory of the senior author (JKF) blinded to treatment allocation and clinical outcomes (JKF has served in angiographic core laboratories including on earlier bivalirudin trials [HERO-1]). However, due to early termination, the senior author adjudicated all TIMI flows except for his procedures, which were adjudicated by author SL. Recurrent myocardial infarction was defined by chest pain lasting ≥30 minutes and accompanied by new ischaemic ECG changes (Q waves >0.04 s, ST-segment depression or ST-segment elevation >1 mm) or further biomarker elevation (troponin I or T > upper reference limit). Stent thrombosis was reported according to the Academic Research Consortium [30]. Target vessel revascularisation was defined as ischaemia-driven repeat revascularisation of the infarct-related artery, requiring repeat PCI or coronary artery bypass graft surgery. Stroke was defined as an ischaemic or haemorrhagic neurological event resulting in loss of neurological function with residual symptoms remaining for at least 24 hours with confirmation on computed tomography or magnetic resonance imaging. Hospitalisation for heart failure was defined as symptoms and signs of heart failure requiring hospital admission.

### Statistical analysis

Our sample size calculation was based on bleeding rates from our previous report of 221 patients undergoing rescue PCI [31]. The projected incidence of any ACUITY bleeding at 90 days was 30% in the heparin ± glycoprotein IIb/IIIa inhibitors arm and 18% in the bivalirudin arm (40% rate reduction). We determined that the enrolment of 205 patients in each study group would provide a power of 80% to detect this difference at a two-sided alpha level of 0.05. The trial was designed for an adaptive sample size re-estimation after recruitment of 70% of the planned total number of patients (n = 280). Due to slow recruitment and futility, the steering committee terminated the trial in 2015 after a blinded interim analysis of 83 patients revealed the event rate of the primary endpoint (11%) was considerably lower than hypothesised (24%). The conditional probability of observing a 40% reduction in the primary endpoint based on this low bleeding event rate would have required enrolling 906 patients to preserve 80% power at a two-sided alpha level of 0.05.

The primary analysis was performed on an intention-to-treat basis and included all randomised patients. In a secondary analysis, we compared the clinical characteristics and outcomes of randomised patients to those screened but not recruited. For the latter patients who were not recruited, retrospective clinical events adjudication was performed by the first author (AF) blinded to treatment allocation.

Categorical variables are presented as numbers (%) and continuous variables as medians (interquartile range [IQR]) or means (± SD). We used the Shapiro-Wilk test to assess the normality of distributions. We plotted Kaplan-Meier cumulative incidence curves for the primary safety endpoint and compared distributions between groups using the log-rank test. Risk ratios (RR) and differences in means, with 95% confidence intervals (CI), are presented for binary and continuous endpoints, respectively. For group comparisons, Pearson’s chi-squared or Fisher’s exact tests were used for binary endpoints, and student’s *t* or the Wilcoxon rank-sum test was used for continuous endpoints. All P-values < 0.05 (two-sided) were considered statistically significant. Statistical analyses were performed using R Studio version 1.1.463 (RStudio, Boston, Massachusetts, USA) and Statistical Product and Service Solutions (SPSS) version 22.0 (IBM, Chicago, Illinois, USA). The data that support the findings of this trial are available from the Supporting Information section of the article (S3 File).

## Results

### Patients and procedures

**Fig 1:**
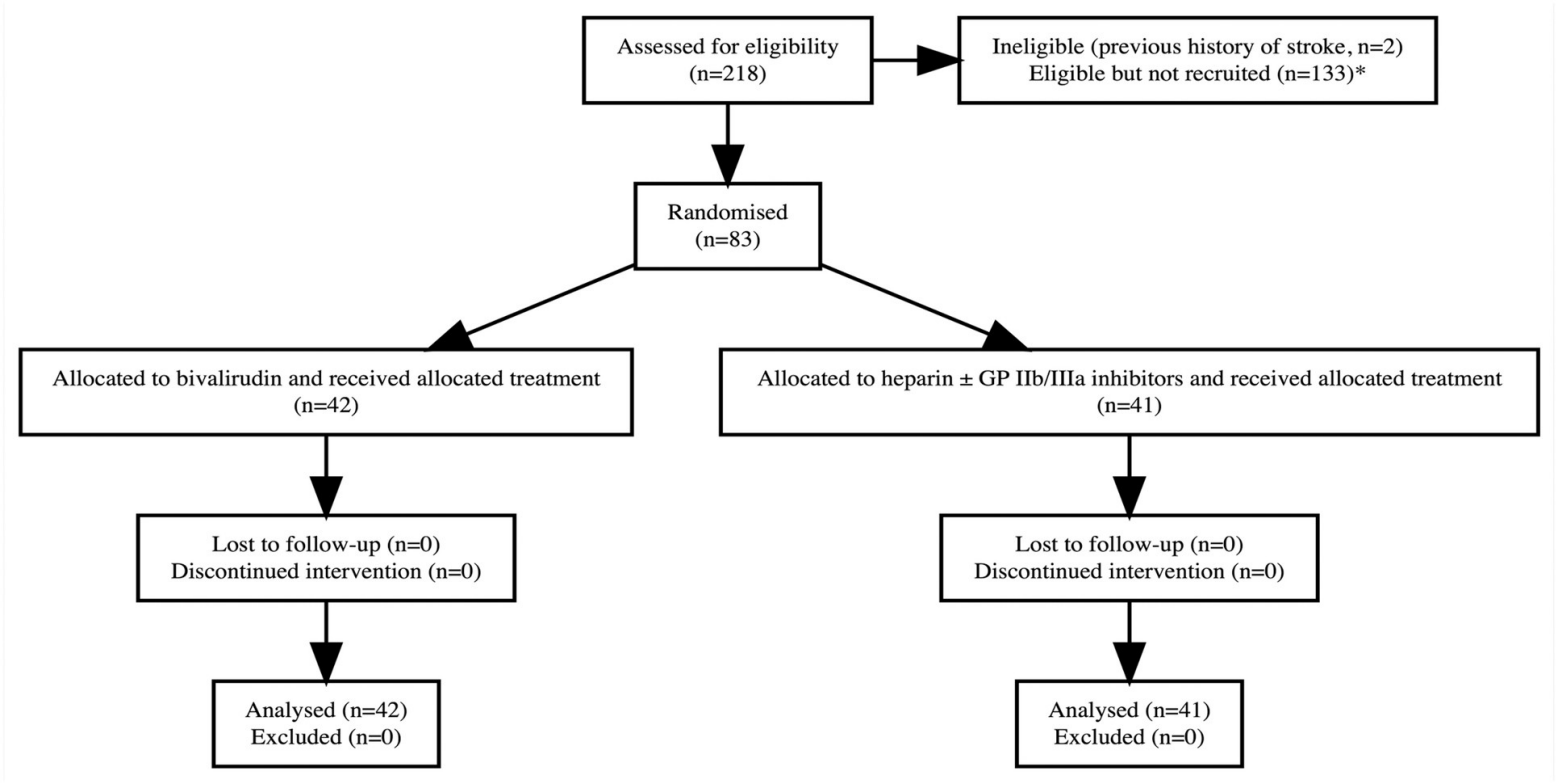
CONSORT diagram of the study population. * Reasons for non-recruitment were not prospectively collected. GP = glycoprotein.

Between August 9, 2010, to July 7, 2015, 83 patients at 9 sites in Australia and New Zealand were randomised to receive bivalirudin (n = 42) or heparin ± glycoprotein IIb/IIIa inhibitors (n = 41) during rescue PCI (Fig 1). Patients randomised to bivalirudin were more often women, had higher rates of diabetes mellitus, dyslipidaemia, pre-hospital fibrinolysis and longer treatment intervals (Table 1). Patients in the heparin ± glycoprotein IIb/IIIa inhibitors group had higher rates of Killip class ≥ 2, previous myocardial infarction and previous PCI. Among all randomised patients, the median age was 63 years (IQR, 55–70), and none had cardiogenic shock before rescue PCI. The infarct-related artery was the left anterior descending in 47% (n = 39) of patients, and stents were used in 95% (n = 70) of patients who underwent PCI (n = 74) (Table 2). All patients received aspirin and clopidogrel before rescue PCI. Glycoprotein IIb/IIIa inhibitors were administered in 4.8% (n = 2) of patients randomised to bivalirudin and 54% (n = 22) of those randomised to heparin. Enoxaparin was administered in 20% (n = 17) of patients as the antithrombin adjunct to fibrinolytic therapy, with no significant differences between the two treatment groups. None received enoxaparin as the antithrombin during PCI. At discharge, P2Y12 inhibitor was prasugrel in 4.9% (n = 2) of patients randomised to bivalirudin and 9.5% (n = 4) of patients in the heparin ± glycoprotein IIb/IIIa inhibitors group.

**Table 1:** Baseline characteristics of the study population.

Variable	Bivalirudin	Heparin ± Glycoprotein IIb/IIIa Inhibitor (n = 41)
	(n = 42)	
Baseline characteristics		
Age (years)	64 (55–68)	62 (55–70)
Female sex	12 (29)	7 (17)
Body mass index (kg/m^2)^	28 (27–33)	29 (25–33)
Past medical history		
Hypertension	26 (62)	24 (59)
Diabetes mellitus	14 (33)	7 (17)
Dyslipidaemia	19 (45)	12 (29)
Smoker	16 (38)	18 (44)
Family history of coronary artery disease	12 (29)	14 (34)
Previous myocardial infarction	0	5 (12)
Previous PCI	1 (2.4)	7 (17)
Previous CABG	1 (2.4)	0
Previous stroke	0	0
Clinical presentation		
Pre-hospital fibrinolysis	6 (14)	4 (9.8)
Cardiogenic shock*	0	0
Cardiac arrest	3 (7.1)	3 (7.3)
Killip class		
1	41 (98)	36 (88)
2	1 (2.4)	5 (12)
3	0	0
4	0	0
Treatment intervals^†^		
Symptom onset-to-lytic (min)	168 (95–257)	136 (100–230)
Symptom onset-to-device (min)	475 (364–588)	395 (281–537)
First medical contact-to-lytic (min)	35 (24–73)	36 (25–52)
First medical contract-to-device (min)	310 (233–443)	240 (205–286)
Baseline investigations		
Haemoglobin (g/L)	154 (140–161)	151 (144–162)
Estimated glomerular filtration rate (ml/min/1.73m^2^)	60 (55–80)	60 (60–78)

Data are presented as n (%) or median (interquartile range).

*Cardiogenic shock was defined as systolic blood pressure <90 mm Hg, lasting ≥1 hour, and end-organ hypoperfusion, with or without mechanical support.

^†^First medical contact was defined as the time of contact with a paramedic or emergency department clinician, whichever was earliest. Device time was defined as the time of the first device used to achieve reperfusion in the infarct-related artery.

CABG = coronary artery bypass graft surgery; PCI = percutaneous coronary intervention.

**Table 2:** Study procedures and medications.

Variable	Bivalirudin	Heparin ± Glycoprotein IIb/IIIa Inhibitor (n = 41)
	(n = 42)	
Radial artery access	17 (41)	22 (54)
Infarct-related artery		
Left main	0	0
Left anterior descending	23 (55)	16 (39)
Circumflex	2 (4.8)	4 (9.8)
Right	16 (38)	21 (51)
Graft	1 (2.4)	0
Initial treatment strategy		
PCI	40 (95)	34 (83)
Stent placement	37 (88)	33 (80)
Balloon angioplasty	3 (7.1)	1 (2.4)
CABG	1 (2.4)	3 (73)
Conservative	1 (2.4)	4 (9.8)
Baseline TIMI flow grade		
0–1	20 (48)	15 (37)
2	10 (24)	11 (27)
3	12 (29)	15 (37)
Post-PCI TIMI flow grade		
0–1	3 (7.1)	1 (2.4)
2	6 (14)	4 (9.8)
3	33 (79)	36 (88)
Antiplatelet agent before coronary angiography		
Aspirin	42 (100)	41 (100)
Clopidogrel	42 (100)	41 (100)
Anti-thrombin before coronary angiography		
Unfractionated heparin	33 (79)	33 (81)
Enoxaparin	9 (21)	8 (20)
Glycoprotein IIb/IIIa inhibitor use		
Planned	1 (2.4)	4 (9.8)
Bailout	1 (2.4)	18 (44)
Medications at hospital discharge		
Aspirin	40 (95)	41 (100)
Clopidogrel	33 (79)	34 (83)
Prasugrel	4 (9.5)	2 (4.9)
Oral anticoagulant	10 (24)	7 (17)
Beta-adrenergic blocker	37 (88)	34 (83)
Statin	38 (90)	37 (90)
Angiotensin-converting enzyme inhibitor	24 (57)	30 (73)
Angiotensin receptor blocker	6 (14)	3 (7.3)
Diuretic	7 (17)	7 (17)

Data are presented as n (%).

PCI = percutaneous coronary intervention; TIMI = Thrombolysis in Myocardial Infarction.

### Safety endpoints

**Fig 2:**
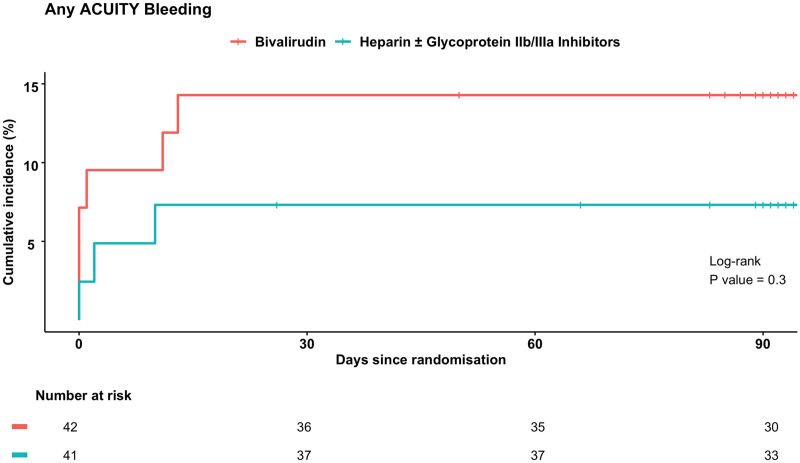
Cumulative incidence curves for the primary endpoint. Cumulative incidence curves for the primary endpoint of any ACUITY bleeding (major and minor) through to 90 days with a table of numbers at risk.

At 90 days, complete follow-up information was available for all patients (n = 83). The primary safety endpoint occurred in 6 patients (14%) in the bivalirudin group and 3 patients (7.3%) in the heparin ± glycoprotein IIb/IIIa inhibitors group (RR, 1.95, 95% CI, 0.52–7.3, *P* = 0.48) (Fig 2 and Table 3). ACUITY major bleeding occurred in 4 patients (9.5%) randomised to bivalirudin and in 2 patients (4.9%) randomised to heparin ± glycoprotein IIb/IIIa inhibitors (RR, 1.95, 95% CI, 0.38–10, *P* = 0.68). No patients suffered TIMI major bleeding. The mean change in the periprocedural haemoglobin level was -7.5% [±15] with bivalirudin and -14% [±17] with heparin ± glycoprotein IIb/IIIa inhibitors (difference in means, -6.5%, 95% CI, -0.83–14, *P* = 0.0067). The blood transfusion rate was similar between the two groups (7.1% for bivalirudin versus 4.9% for heparin ± glycoprotein IIb/IIIa inhibitors, RR, 1.46, 95% CI, 0.26–8.3, *P*>0.99). Death at 90 days occurred in 1 patient (2.4%) randomised to bivalirudin and in 1 patient (2.4%) randomised to heparin ± glycoprotein IIb/IIIa inhibitors. The incidence of other adverse clinical events was low, with no differences between the two groups.

**Table 3:** Clinical endpoints at 90 days.

Outcome	Bivalirudin	Heparin ± Glycoprotein IIb/IIIa Inhibitor	Risk Ratio or Difference in Means (95% CI)	*P*-Value
	(n = 42)	(n = 41)		
Safety endpoints				
Any ACUITY bleeding	6 (14)	3 (7.3)	1.95 (0.52–7.3)	0.48
Major	4 (9.5)	2 (4.9)	1.95 (0.38–10)	0.68
Minor	2 (4.8)	1 (2.4)	1.95 (0.18–21)	>0.99
Any TIMI bleeding	6 (14)	3 (7.3)	1.95 (0.52–7.3)	0.48
Major	0	0	-	-
Minor	2 (4.8)	0	-	0.49
Minimal	4 (9.5)	3 (7.3)	1.30 (0.31–5.5)	>0.99
Intracranial bleeding	0	0	-	-
Blood transfusion	3 (7.1)	2 (4.9)	1.46 (0.26–8.3)	>0.99
Per cent change in haemoglobin level*	-7.5 (±15)	-14 (±17)	-6.5 (-0.83–14)	0.0067
Efficacy endpoints				
Peak troponin I or T divided by URL^†^	730 (±675)	984 (±1585)	254 (-283-794)	0.86
Selvester QRS score^‡^				
ECGs suitable for analysis	38 (90)	33 (80)	-	0.20
Final QRS score^‡^	6.4 (±3.3)	6.5 (±3.2)	0.1 (-1.4–1.6)	0.85
ST-segment recovery^§^				
ECGs suitable for analysis	37 (88)	36 (88)	-	>0.99
Post-fibrinolysis				
Complete (>70%)	4 (11)	1 (2.8)	3.9 (0.46–33)	0.36
Partial (30–70%)	5 (14)	13 (36)	0.37 (0.15–0.94)	0.025
None (<30%)	28 (76)	22 (61)	1.2 (0.90–1.7)	0.18
Post-PCI				
Complete (>70%)	17 (46)	27 (75)	0.61 (0.41–0.91)	0.011
Partial (30–70%)	13 (35)	6 (17)	2.1 (0.90–4.9)	0.072
None (<30%)	7 (19)	3 (8.3)	2.3 (0.64–8.1)	0.31
Other clinical endpoints				
Death	1 (2.4)	1 (2.4)	0.98 (0.063–15)	>0.99
Stroke	0	0	-	-
Recurrent myocardial infarction	2 (4.8)	1 (2.4)	1.95 (0.18–21)	>0.99
Stent thrombosis	1 (2.4)	0	-	>0.99
Target vessel revascularisation	3 (7.1)	0	-	0.24
PCI	1 (2.4)	0	-	>0.99
CABG	2 (4.8)	0	-	0.49
Heart failure hospitalisation	1 (2.4)	0	-	>0.99

Data are presented as n (%) or mean (± SD).

* Change in haemoglobin level is expressed as per cent change between the level before coronary angiography and the next day with adjustment for units of red blood cells transfused.

^†^ Peak troponin level was divided by the upper reference limit of the corresponding assay.

^‡^ Performed on pre-discharge ECGs acquired a median time of 2 days after PCI (IQR, 1–4).

^§^ ST-segment recovery is expressed as a per cent change from baseline in the lead with maximum ST-segment elevation and presented as n (per cent of ECGs analysed). ECGs were acquired post-fibrinolysis at a median time of 71 minutes (IQR, 61–97) and post-PCI at a median time of 32 minutes (IQR, 14–49).

ACUITY = Acute Catheterization and Urgent Intervention Triage Strategy; CABG = coronary artery bypass graft surgery; CI = confidence interval; ECG = electrocardiogram; PCI = percutaneous coronary intervention; TIMI = Thrombolysis in Myocardial Infarction; URL = upper reference limit.

### Efficacy endpoints

Infarct size was similar between the two groups as measured by peak troponin level and the Selvester QRS score (mean peak troponin I or T level / upper reference limit, 730 [±675] for bivalirudin versus 984 [±1585] for heparin, difference in means, 254, 95% CI, -283-794, *P* = 0.86; mean QRS score, 6.4 [±3.3] for bivalirudin versus 6.5 [±3.2] for heparin ± glycoprotein IIb/IIIa inhibitors, difference in means, 0.1, 95% CI, -1.4–1.6, *P* = 0.85) (Table 3). The rate of complete (≥70%) ST-segment recovery post-PCI was higher in patients randomised to heparin ± glycoprotein IIb/IIIa inhibitors compared with bivalirudin (75% versus 46%, RR [bivalirudin relative to heparin], 0.61, 95% CI, 0.41–0.91, *P* = 0.011).

### Secondary analysis

During the study period, 135 patients at the 2 highest recruiting sites underwent rescue PCI but were not recruited. Of these, 2 patients met the study exclusion criteria (previous history of stroke). Reasons for non-recruitment were not prospectively collected and were not known for the remaining patients. Clinical characteristics and outcomes of non-randomised patients are shown in S1-S3 Tables in S1 File. Among non-randomised patients, 82% (n = 110) received heparin (45% with glycoprotein IIb/IIIa inhibitors) and 19% (n = 25) received bivalirudin (none received glycoprotein IIb/IIIa inhibitors) during PCI. Compared with randomised patients, non-randomised patients had a higher incidence of pre-PCI cardiogenic shock, pre-hospital fibrinolysis, pre-PCI TIMI flow grade 0–1 in the infarct-related artery and shorter symptom onset-to-lytic, symptom onset-to-device and FMC-to-lytic times. There was no significant difference in any ACUITY bleeding at 90 days between randomised and non-randomised patients; however, there was a higher incidence of ACUITY minor bleeding at 90 days in non-randomised patients.

## Discussion

In this open-label, multi-centre, randomised trial, we assigned patients with STEMI undergoing rescue PCI to bivalirudin or heparin ± glycoprotein IIb/IIIa inhibitors. After five years, the trial was terminated due to slow recruitment and futility. The main findings are as follows: (1) we were unable to show significant differences in ACUITY bleeding at 90 days and infarct size as measured by peak troponin levels between bivalirudin and heparin ± glycoprotein IIb/IIIa inhibitors. (2) However, there was a smaller decrease in the periprocedural haemoglobin level among those assigned to bivalirudin compared to heparin ± glycoprotein IIb/IIIa inhibitors. (3) The rate of complete (≥70%) ST-segment recovery post-PCI was higher in patients randomised to heparin ± glycoprotein IIb/IIIa inhibitors compared with bivalirudin.

Study-level meta-analyses have shown that in patients with STEMI undergoing primary PCI, periprocedural bivalirudin compared with heparin ± glycoprotein IIb/IIIa inhibitors reduces the risk of major bleeding, increases the risk of acute stent thrombosis, and in some studies, a short-term mortality benefit was observed [10, 12, 32, 33]. Additionally, a recent individual patient-level meta-analysis echoed the latter findings and confirmed a reduced risk of death at 30 days for bivalirudin compared with heparin ± glycoprotein IIb/IIIa inhibitors in patients with STEMI undergoing primary PCI [34, 35]. However, individual randomised controlled trials have reported conflicting results reflecting the considerable heterogeneity between trials. Important sources of heterogeneity include variability in: (1) rates of glycoprotein IIb/IIIa inhibitor use in heparin arms, including planned versus bailout use, (2) type of oral antiplatelets (thienopyridines versus P2Y_12_ inhibitors), (3) unfractionated heparin use before randomisation and the procedural dose, (4) post-PCI bivalirudin infusion regimen (including dose and duration), (5) radial access rates and (6) clinical presentation of the study population (type of ACS and high-risk patient subgroups).

Glycoprotein IIb/IIIa inhibitors and oral antiplatelets can modify the ischaemic and bleeding risk in patients with ACS undergoing PCI [36, 37]. In trials examining bivalirudin in the setting of ACS, oral antiplatelets have shifted from predominantly thienopyridine-based (clopidogrel) [13–15, 18] to the more potent P2Y_12_-based (prasugrel or ticagrelor) [16, 17, 20, 38] regimens. This shift has occurred in parallel with an increase in the use of radial access and a decrease in the use of upstream glycoprotein IIb/IIIa inhibitors [21, 37]. The interplay between these effect modifiers has probably contributed to heterogeneity in the results [9]. Initial trials mandated glycoprotein IIb/IIIa inhibitors in the heparin arm, which may have increased the bleeding risk favouring bivalirudin [13–15]. Subsequent trials used glycoprotein IIb/IIIa inhibitors selectively, with some reporting no bleeding reduction with bivalirudin [17, 38], while in others, a reduction in major bleeding was observed irrespective of glycoprotein IIb/IIIa inhibitor use [18, 21, 39]. Considering the totality of the evidence, the bleeding advantage observed with bivalirudin is likely independent of planned glycoprotein IIb/IIIa inhibitor use in the heparin arm, as confirmed in a recent individual patient-level meta-analysis [34, 35].

Glycoprotein IIb/IIIa inhibitors were used in 54% of patients in the RAFT trial, almost entirely in the heparin arm. This rate is lower than we observed in an earlier decade when there was almost 80% use of these agents and bleeding rates were higher, an era when rescue PCI was almost entirely performed via femoral access [31]. While we could not detect any significant differences in the primary endpoint bleeding between the treatment arms, we observed a smaller decrease in the periprocedural haemoglobin level with bivalirudin, though its significance in the context of premature termination of the trial is speculative.

The use of a high-dose post-PCI bivalirudin infusion (1.75 mg/kg/hr ≤ 4 hours) is an important factor influencing the risk of acute stent thrombosis in patients with STEMI undergoing primary PCI [40]. A post-PCI bivalirudin infusion for 4 hours was protocol-recommended in the RAFT trial. In initial trials without a post-PCI bivalirudin infusion or a low-dose infusion (0.25 mg/kg/hr), an increased risk of acute stent thrombosis in the bivalirudin arm was observed [14, 16, 17]. The increased risk of stent thrombosis has been primarily attributed to the short half-life of bivalirudin in the setting of delayed antiplatelet activity of upstream oral agents. Subsequent meta-analyses and trials have shown that a high-dose post-PCI bivalirudin infusion mitigates the risk of acute stent thrombosis while maintaining a reduction in major bleeding [12, 18, 34, 35, 41–43].

The procedural arterial access site potentially modifies the bleeding benefits seen with bivalirudin. A meta-analysis in 2016 by Mina et al. suggested that bivalirudin reduces bleeding risk only with femoral access [44]. Furthermore, in a recent updated meta-analysis by Kheiri et al. examining bivalirudin in patients undergoing transradial PCI, bivalirudin compared with heparin ± glycoprotein IIb/IIIa inhibitors was associated with a reduced risk of net adverse clinical events; however, there was no significant difference in the risk of major bleeding at 30 days [45]. A significant reduction in major bleeding was only observed compared with heparin + planned glycoprotein IIb/IIIa inhibitors [45]. However, most trials examined in these meta-analyses did not randomise patients according to procedural access, and there may have been confounding factors that were unaccounted for [44, 45]. MATRIX was the only trial that allocated access by randomisation (50% radial access) and showed that the bleeding benefit with bivalirudin was irrespective of access site, with reductions in access site-related and non-access site-related bleeding [21]. Similarly, a reduction in major bleeding with bivalirudin was observed in BRIGHT (Bivalirudin in Acute Myocardial Infarction vs Heparin and GPI Plus Heparin Trial) (78% radial access) and EUROMAX (European Ambulance Acute Coronary Syndrome Angiography Trial) (47% radial access) [16, 18]. In contrast, VALIDATE-SWEDEHEART (Bivalirudin versus Heparin in ST-Segment and Non–ST-Segment Elevation Myocardial Infarction in Patients on Modern Antiplatelet Therapy in the Swedish Web System for Enhancement and Development of Evidence-based Care in Heart Disease Evaluated according to Recommended Therapies Registry Trial) (90% radial access) and HEAT-PPCI (How Effective are Antithrombotic Therapies in Primary Percutaneous Coronary Intervention) (81% radial access) showed no difference in major bleeding with bivalirudin compared with heparin ± glycoprotein IIb/IIIa inhibitors [17, 38]. The bleeding advantage of bivalirudin over heparin ± glycoprotein IIb/IIIa inhibitors is likely independent of procedural access based on the recent individual patient-level meta-analysis reported by Stone et al., which adjusted for procedural access and other clinically relevant covariates [34, 35].

Certain subgroups of patients undergoing PCI are at increased risk of bleeding complications and may derive more benefit from the use of periprocedural bivalirudin [37, 46, 47]. In a post-hoc analysis of the MATRIX trial, patients with cardiogenic shock and cardiac arrest derived greater benefit from using bivalirudin over heparin ± glycoprotein IIb/IIIa inhibitors [46]. Patients undergoing rescue PCI are a high-risk group and are at risk of bleeding complications due to the concomitant administration of fibrinolytic therapy, antithrombins and antiplatelet agents. In this regard, this patient group may derive a greater benefit from the bleeding advantage of bivalirudin. However, although our study population included rescue PCI patients, none had presented with cardiogenic shock, very few patients presented with advanced Killip class, and we observed an overall low bleeding event rate. Our sample size calculation was based on our previous report of 221 patients undergoing rescue PCI, 99% via femoral access, and with 78% upstream glycoprotein IIb/IIIa inhibitor use [31]. Thus, the combination of the low-risk features of the trial population and the reduction of femoral access and lower glycoprotein IIb/IIIa inhibitor use likely contributed to the observed lower than hypothesised bleeding event rate.

We found a higher rate of complete ST-segment recovery post-PCI with heparin ± glycoprotein IIb/IIIa inhibitors than bivalirudin. However, in post-hoc analyses of the HORIZONS-AMI (Harmonizing Outcomes with Revascularisation and Stents in Acute Myocardial Infarction) and EUROMAX (European Ambulance Acute Coronary Syndrome Angiography trial) trials, which randomised patients to bivalirudin or heparin ± glycoprotein IIb/IIIa inhibitors in primary PCI, no difference in the rate of complete ST-segment recovery was observed between the two treatment arms [48, 49]. Given that the RAFT trial was not powered to assess the endpoint of ST-segment recovery, this observed difference was likely due to type I error.

For a large proportion of patients with STEMI, primary PCI cannot be performed promptly due to logistical barriers or geographic disparities [50]. Pharmaco-invasive PCI remains an important reperfusion strategy, and our preliminary report found a lower mortality than with all-comers primary PCI [51]. However, rescue PCI is associated with a higher risk of bleeding complications and trials evaluating different adjunctive therapies in the setting of contemporary pharmaco-invasive PCI are lacking. The TREAT (Ticagrelor in Patients With ST-Elevation Myocardial Infarction Treated With Pharmacological Thrombolysis) trial was the most recent trial in this domain and compared ticagrelor versus clopidogrel in patients < 75 years of age treated with a pharmaco-invasive strategy [52]. Ticagrelor was found to be non-inferior to clopidogrel for TIMI major bleeding at 30 days [52]. The EARLY-MYO Trial (Early Routine Catheterization After Alteplase Fibrinolysis Versus Primary PCI in Acute ST-Segment–Elevation Myocardial Infarction) compared a pharmaco-invasive strategy with half-dose tenecteplase to primary PCI in patients < 75 years of age and reported more complete epicardial and myocardial reperfusion in patients treated with a pharmaco-invasive approach with no differences in the rates of major bleeding [53]. The currently ongoing large-scale STREAM-2 (Strategic Reperfusion Early After Myocardial Infarction) (NCT02777580) trial, in which pharmaco-invasive PCI with half-dose tenecteplase is compared to primary PCI in patients > 60 years of age, will provide further insights on the optimal pharmaco-invasive strategy in elderly patients who cannot undergo timely primary PCI.

### Limitations

This trial has several important limitations. First, premature termination and the consequent small sample size did not allow us to detect significant differences in the primary endpoint between the two treatment groups. Thus, the lower periprocedural reduction in periprocedural haemoglobin must be considered hypothesis-generating. Second, the open-label design and the randomisation process likely contributed to selection bias towards low-risk patients, based on our contemporaneous group of unselected patients undergoing rescue PCI. Randomisation occurred after the decision to perform rescue PCI, and in patients with haemodynamic instability or severe pain, the logistics of verbal consent and randomisation may have influenced patient selection by trial investigators. Third, due to lack of funding, we modified the primary efficacy biomarker endpoint to peak troponin I or T level, which we have previously shown to correlate with the original pre-specified primary efficacy endpoint of the area under the curve of creatine kinase-MB [23]. Fourth, though the study population is not contemporary as the trial enrolled patients between 2010 and 2015, second-generation drug-eluting stents were used. Finally, clinical events were site-reported, and the analysis of ECGs was performed by the first author and not at a core laboratory.

## Conclusions

Among patients undergoing rescue PCI, we were unable to demonstrate significant differences in ACUITY bleeding at 90 days or infarct size as measured by peak troponin level between bivalirudin and heparin ± glycoprotein IIb/IIIa inhibitors. There was a smaller decrease in the periprocedural haemoglobin level with bivalirudin than heparin ± glycoprotein IIb/IIIa inhibitors. The rate of complete (≥70%) ST-segment recovery post-PCI was higher in patients randomised to heparin ± glycoprotein IIb/IIIa inhibitors compared with bivalirudin. However, interpretation of the results is limited by the premature termination of the trial.

## Supporting information

S1 Fig Trial design. Flow diagram of study design. Patients treated with fibrinolytic therapy and referred for urgent angiography because of failure to achieve 50% ST-segment recovery at 60–90 minutes were randomised to receive either bivalirudin or heparin ± glycoprotein IIb/IIIa inhibitors if they were suitable for percutaneous coronary intervention. The primary safety endpoint was any ACUITY bleeding at 90 days. Infarct size was measured by peak troponin I or T levels and expressed as a multiple of the upper reference limit of the corresponding assay. ACUITY = Acute Catheterization and Urgent Intervention Triage Strategy; PCI = percutaneous coronary intervention; STEMI = ST-segment elevation myocardial infarction.
(TIF)

S1 File Secondary analysis comparing clinical characteristics and outcomes of randomised and non-randomised patients. (DOCX)

S2 File Trial protocol. RAFT trial protocol.
(PDF)

S3 File Trial data. RAFT trial dataset.
(XLSX)

S1 Checklist RAFT trial CONSORT checklist. (DOC)

## References

[1] Ellis SG, Da Silva ER, Spaulding CM, Nobuyoshi M, Weiner B, Talley JD. Review of immediate angioplasty after fibrinolytic therapy for acute myocardial infarction: insights from the RESCUE I, RESCUE II, and other contemporary clinical experiences. Am Heart J. 2000;139: 1046–1053. doi: 10.1067/mhj.2000.106624 10827386

[2] Gershlick AH, Stephens-Lloyd A, Hughes S, Abrams KR, Stevens SE, Uren NG, et al. Rescue angioplasty after failed thrombolytic therapy for acute myocardial infarction. N Engl J Med. 2005;353: 2758–2768. doi: 10.1056/NEJMoa050849 16382062

[3] Wijeysundera HC, Vijayaraghavan R, Nallamothu BK, Foody JM, Krumholz HM, Phillips CO, et al. Rescue angioplasty or repeat fibrinolysis after failed fibrinolytic therapy for ST-segment myocardial infarction: a meta-analysis of randomized trials. J Am Coll Cardiol. 2007;49: 422–430. doi: 10.1016/j.jacc.2006.09.033 17258087

[4] Welsh RC, Van de Werf F, Westerhout CM, Goldstein P, Gershlick AH, Wilcox RG, et al. Outcomes of a pharmacoinvasive strategy for successful versus failed fibrinolysis and primary percutaneous intervention in acute myocardial infarction (from the STrategic Reperfusion Early After Myocardial Infarction [STREAM] study). Am J Cardiol. 2014;114: 811–819. doi: 10.1016/j.amjcard.2014.06.011 25108302

[5] Lidón RM, Théroux P, Lespérance J, Adelman B, Bonan R, Duval D, et al. A pilot, early angiographic patency study using a direct thrombin inhibitor as adjunctive therapy to streptokinase in acute myocardial infarction. Circulation. 1994;89: 1567–1572. doi: 10.1161/01.cir.89.4.1567 8149522

[6] Théroux P, Pérez-Villa F, Waters D, Lespérance J, Shabani F, Bonan R. Randomized double-blind comparison of two doses of Hirulog with heparin as adjunctive therapy to streptokinase to promote early patency of the infarct-related artery in acute myocardial infarction. Circulation. 1995;91: 2132–2139. doi: 10.1161/01.cir.91.8.2132 7697841

[7] White HD, Aylward PE, Frey MJ, Adgey AA, Nair R, Hillis WS, et al. Randomized, double-blind comparison of hirulog versus heparin in patients receiving streptokinase and aspirin for acute myocardial infarction (HERO). Hirulog Early Reperfusion/Occlusion (HERO) Trial Investigators. Circulation. 1997;96: 2155–2161. doi: 10.1161/01.cir.96.7.2155 9337184

[8] White H, Hirulog and Early Reperfusion or Occlusion (HERO)-2 Trial Investigators. Thrombin-specific anticoagulation with bivalirudin versus heparin in patients receiving fibrinolytic therapy for acute myocardial infarction: the HERO-2 randomised trial. Lancet. 2001;358: 1855–1863. doi: 10.1016/s0140-6736(01)06887-8 11741625

[9] Capodanno D, Angiolillo DJ. Pooling the Evidence at the Patient Level: End of the Bivalirudin Saga? Thromb Haemost. 2020;120: 191–193. doi: 10.1055/s-0039-3400743 31899923

[10] Capodanno D, Gargiulo G, Capranzano P, Mehran R, Tamburino C, Stone GW. Bivalirudin versus heparin with or without glycoprotein IIb/IIIa inhibitors in patients with STEMI undergoing primary PCI: An updated meta-analysis of 10,350 patients from five randomized clinical trials. Eur Heart J Acute Cardiovasc Care. 2016;5: 253–262. 25746943 10.1177/2048872615572599

[11] Shah R, Rogers KC, Matin K, Askari R, Rao SV. An updated comprehensive meta-analysis of bivalirudin vs heparin use in primary percutaneous coronary intervention. Am Heart J. 2016;171: 14–24. doi: 10.1016/j.ahj.2015.10.006 26699596

[12] Fahrni G, Wolfrum M, De Maria GL, Banning AP, Benedetto U, Kharbanda RK. Prolonged High-Dose Bivalirudin Infusion Reduces Major Bleeding Without Increasing Stent Thrombosis in Patients Undergoing Primary Percutaneous Coronary Intervention: Novel Insights From an Updated Meta-Analysis. J Am Heart Assoc. 2016;5: e003515. doi: 10.1161/JAHA.116.003515 27451466 PMC5015387

[13] Stone GW, McLaurin BT, Cox DA, Bertrand ME, Lincoff AM, Moses JW, et al. Bivalirudin for patients with acute coronary syndromes. N Engl J Med. 2006;355: 2203–2216. doi: 10.1056/NEJMoa062437 17124018

[14] Stone GW, Witzenbichler B, Guagliumi G, Peruga JZ, Brodie BR, Dudek D, et al. Bivalirudin during primary PCI in acute myocardial infarction. N Engl J Med. 2008;358: 2218–2230. doi: 10.1056/NEJMoa0708191 18499566

[15] Kastrati A, Neumann F-J, Schulz S, Massberg S, Byrne RA, Ferenc M, et al. Abciximab and heparin versus bivalirudin for non-ST-elevation myocardial infarction. N Engl J Med. 2011;365: 1980–1989. doi: 10.1056/NEJMoa1109596 22077909

[16] Steg PG, van ‘t Hof A, Hamm CW, Clemmensen P, Lapostolle F, Coste P, et al. Bivalirudin started during emergency transport for primary PCI. N Engl J Med. 2013;369: 2207–2217. doi: 10.1056/NEJMoa1311096 24171490

[17] Shahzad A, Kemp I, Mars C, Wilson K, Roome C, Cooper R, et al. Unfractionated heparin versus bivalirudin in primary percutaneous coronary intervention (HEAT-PPCI): an open-label, single centre, randomised controlled trial. Lancet. 2014;384: 1849–1858. doi: 10.1016/S0140-6736(14)60924-7 25002178

[18] Han Y, Guo J, Zheng Y, Zang H, Su X, Wang Y, et al. Bivalirudin vs heparin with or without tirofiban during primary percutaneous coronary intervention in acute myocardial infarction: the BRIGHT randomized clinical trial. JAMA. 2015;313: 1336–1346. doi: 10.1001/jama.2015.2323 25775052

[19] Erlinge D, Omerovic E, Fröbert O, Linder R, Danielewicz M, Hamid M, et al. Bivalirudin versus Heparin Monotherapy in Myocardial Infarction. N Engl J Med. 2017;377: 1132–1142. doi: 10.1056/NEJMoa1706443 28844201

[20] Valgimigli M, Frigoli E, Leonardi S, Rothenbühler M, Gagnor A, Calabrò P, et al. Bivalirudin or Unfractionated Heparin in Acute Coronary Syndromes. N Engl J Med. 2015;373: 997–1009. doi: 10.1056/NEJMoa1507854 26324049

[21] Gargiulo G, Carrara G, Frigoli E, Vranckx P, Leonardi S, Ciociano N, et al. Bivalirudin or Heparin in Patients Undergoing Invasive Management of Acute Coronary Syndromes. J Am Coll Cardiol. 2018;71: 1231–1242. doi: 10.1016/j.jacc.2018.01.033 29544607

[22] Nguyen TL, Phan JAK, Hee L, Moses DA, Otton J, Terreblanche OD, et al. High-sensitivity troponin T predicts infarct scar characteristics and adverse left ventricular function by cardiac magnetic resonance imaging early after reperfused acute myocardial infarction. Am Heart J. 2015;170: 715–725.e2. doi: 10.1016/j.ahj.2015.06.022 26386795

[23] Shugman IM, Diu P, Gohil J, Kadappu KK, Leung M, Lo S, et al. Evaluation of troponin T criteria for periprocedural myocardial infarction in patients with acute coronary syndromes. Am J Cardiol. 2011;107: 863–870. doi: 10.1016/j.amjcard.2010.11.007 21376928

[24] Bovill EG, Terrin ML, Stump DC, Berke AD, Frederick M, Collen D, et al. Hemorrhagic events during therapy with recombinant tissue-type plasminogen activator, heparin, and aspirin for acute myocardial infarction. Results of the Thrombolysis in Myocardial Infarction (TIMI), Phase II Trial. Ann Intern Med. 1991;115: 256–265. doi: 10.7326/0003-4819-115-4-256 1906692

[25] Loring Z, Chelliah S, Selvester RH, Wagner G, Strauss DG. A detailed guide for quantification of myocardial scar with the Selvester QRS score in the presence of electrocardiogram confounders. J Electrocardiol. 2011;44: 544–554. doi: 10.1016/j.jelectrocard.2011.06.008 21872001 PMC3164517

[26] French JK, Andrews J, Manda SOM, Stewart RAH, McTigue JJC, White HD. Early ST-segment recovery, infarct artery blood flow, and long-term outcome after acute myocardial infarction. Am Heart J. 2002;143: 265–271. doi: 10.1067/mhj.2002.120147 11835029

[27] McLaughlin MG, Stone GW, Aymong E, Gardner G, Mehran R, Lansky AJ, et al. Prognostic utility of comparative methods for assessment of ST-segment resolution after primary angioplasty for acute myocardial infarction: the Controlled Abciximab and Device Investigation to Lower Late Angioplasty Complications (CADILLAC) trial. J Am Coll Cardiol. 2004;44: 1215–1223. doi: 10.1016/j.jacc.2004.06.053 15364322

[28] Wiesen AR, Hospenthal DR, Byrd JC, Glass KL, Howard RS, Diehl LF. Equilibration of hemoglobin concentration after transfusion in medical inpatients not actively bleeding. Ann Intern Med. 1994;121: 278–230. doi: 10.7326/0003-4819-121-4-199408150-00009 8037410

[29] Schröder R, Dissmann R, Brüggemann T, Wegscheider K, Linderer T, Tebbe U, et al. Extent of early ST segment elevation resolution: a simple but strong predictor of outcome in patients with acute myocardial infarction. J Am Coll Cardiol. 1994;24: 384–391. doi: 10.1016/0735-1097(94)90292-5 8034872

[30] Garcia-Garcia HM, McFadden EP, Farb A, Mehran R, Stone GW, Spertus J, et al. Standardized End Point Definitions for Coronary Intervention Trials: The Academic Research Consortium-2 Consensus Document. Circulation. 2018;137: 2635–2650. doi: 10.1161/CIRCULATIONAHA.117.029289 29891620

[31] Shugman IM, Hsieh V, Cheng S, Parikh D, Tobing D, Wouters N, et al. Safety and efficacy of rescue angioplasty for ST-elevation myocardial infarction with high utilization rates of glycoprotein IIb/IIIa inhibitors. Am Heart J. 2012;163: 649–656.e1. doi: 10.1016/j.ahj.2012.01.014 22520531

[32] Bangalore S, Toklu B, Kotwal A, Volodarskiy A, Sharma S, Kirtane AJ, et al. Anticoagulant therapy during primary percutaneous coronary intervention for acute myocardial infarction: a meta-analysis of randomized trials in the era of stents and P2Y12 inhibitors. BMJ. 2014;349: g6419. doi: 10.1136/bmj.g6419 25389143 PMC4227311

[33] Navarese EP, Schulze V, Andreotti F, Kowalewski M, Kołodziejczak M, Kandzari DE, et al. Comprehensive meta-analysis of safety and efficacy of bivalirudin versus heparin with or without routine glycoprotein IIb/IIIa inhibitors in patients with acute coronary syndrome. JACC Cardiovasc Interv. 2015;8: 201–213. doi: 10.1016/j.jcin.2014.10.003 25616926

[34] Bikdeli B, McAndrew T, Crowley A, Chen S, Mehdipoor G, Redfors B, et al. Individual Patient Data Pooled Analysis of Randomized Trials of Bivalirudin versus Heparin in Acute Myocardial Infarction: Rationale and Methodology. Thromb Haemost. 2020;120: 348–362. doi: 10.1055/s-0039-1700872 31820428 PMC7040882

[35] Stone GW. Individual Patient Data Pooled Analysis of Randomized Trials of Bivalirudin Versus Heparin in Acute Myocardial Infarction. In: TCTMD.com [Internet]. 2020 [cited 23 Aug 2021]. https://www.tctmd.com/slide/individual-patient-data-pooled-analysis-randomized-trials-bivalirudin-versus-heparin-acute 10.1055/s-0039-1700872 PMC7040882 31820428

[36] Gargiulo G, Esposito G, Avvedimento M, Nagler M, Minuz P, Campo G, et al. Cangrelor, Tirofiban, and Chewed or Standard Prasugrel Regimens in Patients With ST-Segment-Elevation Myocardial Infarction: Primary Results of the FABOLUS-FASTER Trial. Circulation. 2020;142: 441–454. doi: 10.1161/CIRCULATIONAHA.120.046928 32795098 PMC7392586

[37] Singh M. Bleeding avoidance strategies during percutaneous coronary interventions. J Am Coll Cardiol. 2015;65: 2225–2238. doi: 10.1016/j.jacc.2015.03.567 25998668

[38] Erlinge D, Koul S, Eriksson P, Scherstén F, Omerovic E, Linder R, et al. Bivalirudin versus heparin in non-ST and ST-segment elevation myocardial infarction-a registry-based randomized clinical trial in the SWEDEHEART registry (the VALIDATE-SWEDEHEART trial). Am Heart J. 2016;175: 36–46. doi: 10.1016/j.ahj.2016.02.007 27179722

[39] Zeymer U, van ‘t Hof A, Adgey J, Nibbe L, Clemmensen P, Cavallini C, et al. Bivalirudin is superior to heparins alone with bailout GP IIb/IIIa inhibitors in patients with ST-segment elevation myocardial infarction transported emergently for primary percutaneous coronary intervention: a pre-specified analysis from the EUROMAX trial. Eur Heart J. 2014;35: 2460–2467. 24849104 10.1093/eurheartj/ehu214 PMC4169872

[40] Valgimigli M, Gargiulo G. Bivalirudin in Current Practice: Melius Abundare Quam Deficere? JACC Cardiovasc Interv. 2016;9: 1321–1323. doi: 10.1016/j.jcin.2016.05.038 27318842

[41] Gargiulo G, Carrara G, Frigoli E, Leonardi S, Vranckx P, Campo G, et al. Post-Procedural Bivalirudin Infusion at Full or Low Regimen in Patients With Acute Coronary Syndrome. J Am Coll Cardiol. 2019;73: 758–774. doi: 10.1016/j.jacc.2018.12.023 30784669

[42] Shah R, Latham SB, Porta JM, Naz A, Matin K, Rao SV. Bivalirudin with a post-procedure infusion versus heparin monotherapy for the prevention of stent thrombosis. Catheter Cardiovasc Interv. 2019;94: 210–215. doi: 10.1002/ccd.28065 30636368

[43] Clemmensen P, Wiberg S, Van’t Hof A, Deliargyris EN, Coste P, Ten Berg J, et al. Acute stent thrombosis after primary percutaneous coronary intervention: insights from the EUROMAX trial (European Ambulance Acute Coronary Syndrome Angiography). JACC Cardiovasc Interv. 2015;8: 214–220. doi: 10.1016/j.jcin.2014.11.002 25616927

[44] Mina GS, Gobrial GF, Modi K, Dominic P. Combined Use of Bivalirudin and Radial Access in Acute Coronary Syndromes Is Not Superior to the Use of Either One Separately: Meta-Analysis of Randomized Controlled Trials. JACC Cardiovasc Interv. 2016;9: 1523–1531. doi: 10.1016/j.jcin.2016.05.023 27491601

[45] Kheiri B, Rao SV, Osman M, Simpson TF, Barbarawi M, Zayed Y, et al. Meta-analysis of bivalirudin versus heparin in transradial coronary interventions. Catheter Cardiovasc Interv. 2020;96: 1240–1248. doi: 10.1002/ccd.28800 32091668

[46] Gargiulo G, Valgimigli M, Sunnåker M, Vranckx P, Frigoli E, Leonardi S, et al. Choice of access site and type of anticoagulant in acute coronary syndromes with advanced Killip class or out-of-hospital cardiac arrest. Rev Esp Cardiol (Engl Ed). 2020. 10.1016/j.rec.2020.01.005 32151464

[47] Gragnano F, Spirito A, Corpataux N, Vaisnora L, Galea R, Gargiulo G, et al. Impact of Clinical Presentation on Bleeding Risk after Percutaneous Coronary Intervention and Implications for the ARC-HBR Definition. EuroIntervention. 2021; EIJ-D-21-00181. doi: 10.4244/EIJ-D-21-00181 34105513 PMC9725019

[48] Farkouh ME, Reiffel J, Dressler O, Nikolsky E, Parise H, Cristea E, et al. Relationship between ST-segment recovery and clinical outcomes after primary percutaneous coronary intervention: the HORIZONS-AMI ECG substudy report. Circ Cardiovasc Interv. 2013;6: 216–223. doi: 10.1161/CIRCINTERVENTIONS.112.000142 23652600

[49] Van’t Hof A, Giannini F, Ten Berg J, Tolsma R, Clemmensen P, Bernstein D, et al. ST-segment resolution with bivalirudin versus heparin and routine glycoprotein IIb/IIIa inhibitors started in the ambulance in ST-segment elevation myocardial infarction patients transported for primary percutaneous coronary intervention: The EUROMAX ST-segment resolution substudy. Eur Heart J Acute Cardiovasc Care. 2017;6: 404–411. 26250825 10.1177/2048872615598633

[50] Aliprandi-Costa B, Morgan L, Snell LC, D Souza M, Kritharides L, French J, et al. ST-Elevation Acute Myocardial Infarction in Australia-Temporal Trends in Patient Management and Outcomes 1999–2016. Heart Lung Circ. 2019;28: 1000–1008. doi: 10.1016/j.hlc.2018.05.191 30006115

[51] Idris H, Yang W, Burgess S, Faour A, McLean A, Sidney Lo S, et al. P333 Late mortality after pharmaco-invasive PCI for STEMI. Eur Heart J. 2019;40. doi: 10.1093/eurheartj/ehz747.0167

[52] Berwanger O, Nicolau JC, Carvalho AC, Jiang L, Goodman SG, Nicholls SJ, et al. Ticagrelor vs Clopidogrel After Fibrinolytic Therapy in Patients With ST-Elevation Myocardial Infarction: A Randomized Clinical Trial. JAMA Cardiol. 2018;3: 391–399. 29525822 10.1001/jamacardio.2018.0612 PMC5875327

[53] Pu J, Ding S, Ge H, Han Y, Guo J, Lin R, et al. Efficacy and Safety of a Pharmaco-Invasive Strategy With Half-Dose Alteplase Versus Primary Angioplasty in ST-Segment-Elevation Myocardial Infarction: EARLY-MYO Trial (Early Routine Catheterization After Alteplase Fibrinolysis Versus Primary PCI in Acute ST-Segment-Elevation Myocardial Infarction). Circulation. 2017;136: 1462–1473. doi: 10.1161/CIRCULATIONAHA.117.030582 28844990

