# Supplementary figures and images for: Reperfusion After Fibrinolytic Therapy (RAFT): An open-label, multi-centre, randomised controlled trial of bivalirudin versus heparin in rescue percutaneous coronary intervention
Source: PLoS One. 2021 Oct 26;16(10):e0259148. doi: 10.1371/journal.pone.0259148 (PMC8547635; doi:10.1371/journal.pone.0259148)

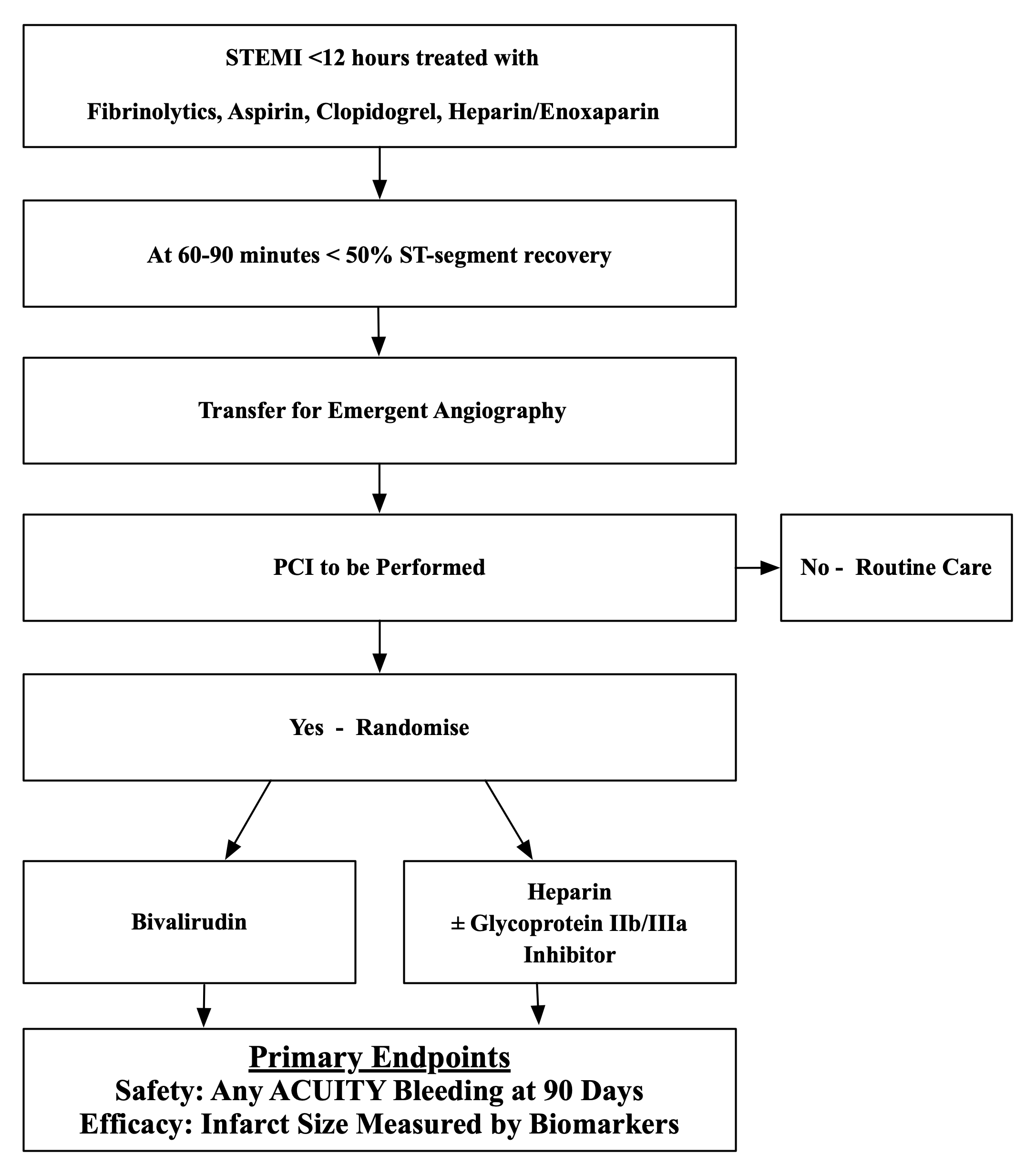

Supplement: S1 Fig — Flow diagram of study design. Patients treated with fibrinolytic therapy and referred for urgent angiography because of failure to achieve 50% ST-segment recovery at 60–90 minutes were randomised to receive either bivalirudin or heparin ± glycoprotein IIb/IIIa inhibitors if they were suitable for percutaneous coronary intervention. The primary safety endpoint was any ACUITY bleeding at 90 days. Infarct size was measured by peak troponin I or T levels and expressed as a multiple of the upper reference limit of the corresponding assay. ACUITY = Acute Catheterization and Urgent Intervention Triage Strategy; PCI = percutaneous coronary intervention; STEMI = ST-segment elevation myocardial infarction. (TIF) [file pone.0259148.s001.tif]
