# Supplementary material for: Reperfusion After Fibrinolytic Therapy (RAFT): An open-label, multi-centre, randomised controlled trial of bivalirudin versus heparin in rescue percutaneous coronary intervention
Source: PLoS One. 2021 Oct 26;16(10):e0259148. doi: 10.1371/journal.pone.0259148 (PMC8547635; doi:10.1371/journal.pone.0259148)
Supplement: S1 File — (DOCX) [file pone.0259148.s002.docx]

**S1 File**

**S1 Table. Baseline characteristics of randomised and non-randomised patients.**

| **Variable** | **Randomised** (n=83) | **Non-Randomised** (n=135) | ***P*-Value** |
| --- | --- | --- | --- |
| Baseline characteristics | | | |
| Age (years) | 63 (55-69) | 59 (52-68) | 0.14 |
| Female sex | 19 (23) | 29 (21) | 0.81 |
| Body mass index (kg/m^2)^ | 28 (26-33) | 29 (26-32) | 0.71 |
| Past medical history | | | |
| Hypertension | 50 (60) | 70 (52) | 0.23 |
| Diabetes mellitus | 21 (25) | 23 (17) | 0.14 |
| Dyslipidaemia | 31 (37) | 53 (39) | 0.78 |
| Smoker | 34 (41) | 58 (43) | 0.77 |
| Family history of coronary artery disease | 26 (31) | 41 (30) | 0.88 |
| Previous myocardial infarction | 5 (6.0) | 9 (6.7) | 0.85 |
| Previous PCI | 8 (9.6) | 11 (8.1) | 0.70 |
| Previous CABG | 1 (1.2) | 2 (1.5) | >0.99 |
| Previous stroke | 0 (0) | 2 (1.5) | 0.53 |
| Clinical presentation | | | |
| Pre-hospital fibrinolysis | 10 (12) | 31 (23) | 0.045 |
| Cardiogenic shock^*^ | 0 (0) | 19 (14) | <0.001 |
| Killip class |  |  |  |
| 1 | 77 (93) | 87 (64) | <0.001 |
| 2 | 6 (7.2) | 27 (20) | 0.011 |
| 3 | 0 (0) | 2 (1.5) | 0.53 |
| 4 | 0 (0) | 19 (14) | <0.001 |
| Treatment intervals^†^ | | | |
| Symptom onset-to-lytic (min) | 146 (96-252) | 95 (60-170) | <0.001 |
| Symptom onset-to-device (min) | 455 (320-586) | 362 (246-510) | 0.016 |
| First medical contact-to-lytic (min) | 35 (25-58) | 29 (20-40) | 0.003 |
| First medical contract-to-device (min) | 261 (208-367) | 264 (180-374) | 0.46 |
| Data are presented as n (%) or median (interquartile range).  ^*^Cardiogenic shock was defined as systolic blood pressure <90 mm Hg, lasting ≥1 hour, and end-organ hypoperfusion, with or without mechanical support.  ^†^First medical contact was defined as time of contact with a paramedic or emergency department clinician, whichever was earliest. Device time was defined as time of the first device used to achieve reperfusion in the infarct-related artery.  CABG = coronary artery bypass graft surgery; PCI = percutaneous coronary intervention. | | | |

**S2 Table. Procedures and medications of randomised and non-randomised patients.**

| **Variable** | **Randomised** (n=83) | **Non-Randomised** (n=135) | ***P*-Value** |
| --- | --- | --- | --- |
| Radial artery access | 39 (47) | 59 (44) | 0.67 |
| Infarct-related artery | | | |
| Left main | 0 | 6 (4.4) | 0.090 |
| Left anterior descending | 39 (47) | 74 (55) | 0.24 |
| Circumflex | 6 (7.2) | 10 (7.4) | 0.96 |
| Right | 37 (45) | 44 (33) | 0.080 |
| Graft | 1 (1.2) | 1 (0.7) | >0.99 |
| Initial treatment strategy | | | |
| PCI | 74 (89) | 135 (100) | <0.001 |
| Stent placement | 70 (84) | 127 (94) | 0.018 |
| Balloon angioplasty | 4 (4.8) | 8 (5.9) | >0.99 |
| Baseline TIMI flow grade | | | |
| 0-1 | 35 (42) | 84 (62) | <0.001 |
| 2 | 21 (25) | 30 (22) | 0.60 |
| 3 | 27 (33) | 21 (16) | 0.030 |
| Post-PCI TIMI flow grade | | | |
| 0-1 | 4 (4.8) | 6 (4.4) | >0.99 |
| 2 | 10 (12) | 13 (9.6) | 0.57 |
| 3 | 69 (83) | 116 (86) | 0.58 |
| P2Y12 inhibitor prior to coronary angiography | | | |
| Clopidogrel | 83 (100) | 131 (97) | 0.30 |
| Prasugrel | 0 | 3 (2.2) | 0.29 |
| Ticagrelor | 0 | 1 (0.7) | >0.99 |
| Anti-thrombin prior to coronary angiography | | | |
| Unfractionated heparin | 41 (49) | 104 (100) | <0.001 |
| Enoxaparin | 8 (9.6) | 31 (23) | 0.013 |
| Bivalirudin use during PCI | 42 (51) | 25 (19) | <0.001 |
| Glycoprotein IIb/IIIa inhibitor use | 24 (29) | 52 (39) | 0.15 |
| Data are presented as n (%).  PCI = percutaneous coronary intervention; TIMI = Thrombolysis in Myocardial Infarction. | | | |

**S3 Table. Clinical outcomes at 90 days of randomised and non-randomised patients.**

| **Variable** | **Randomised** (n=83) | **Non-Randomised** (n=135) | ***P*-Value** |
| --- | --- | --- | --- |
| Safety endpoints | | | |
| Any ACUITY bleeding | 9 (11) | 25 (19) | 0.13 |
| Major | 6 (7.2) | 6 (4.4) | 0.38 |
| Minor | 3 (3.6) | 19 (14) | 0.013 |
| Any TIMI bleeding | 9 (11) | 25 (19) | 0.13 |
| Major | 0 (0) | 3 (2.2) | 0.29 |
| Minor | 2 (2.4) | 7 (5.2) | 0.49 |
| Minimal | 7 (8.4) | 15 (11) | 0.52 |
| Intracranial bleeding | 0 (0) | 1 (0.7) | >0.99 |
| Blood transfusion | 5 (6.0) | 5 (3.7) | 0.51 |
| Efficacy endpoints | | | |
| Peak troponin I or T divided by URL^*^ | 855 (±1212) | 1100 (±1283) | 0.14 |
| Selvester QRS score^†^ |  |  |  |
| ECGs suitable for analysis | 71 (86) | 96 (71) | 0.015 |
| Final QRS score^†^ | 6.5 (3.2) | 7.2 (3.0) | 0.14 |
| ST-segment recovery^‡^ |  |  |  |
| ECGs suitable for analysis | 73 (88) | 91 (67) | <0.001 |
| Post-fibrinolysis |  |  |  |
| Complete (>70%) | 5 (6.8) | 11 (11) | 0.31 |
| Partial (30-70%) | 18 (25) | 30 (31) | 0.35 |
| None (<30%) | 50 (68) | 55 (57) | 0.14 |
| Post-PCI |  |  |  |
| Complete (>70%) | 44 (60) | 50 (49) | 0.12 |
| Partial (30-70%) | 19 (26) | 39 (38) | 0.10 |
| None (<30%) | 10 (14) | 14 (14) | 0.98 |
| Other clinical endpoints | | | |
| Death | 2 (2.4) | 11 (8.1) | 0.14 |
| Stroke | 0 (0) | 1 (0.7) | >0.99 |
| Recurrent myocardial infarction | 3 (3.6) | 3 (2.2) | 0.68 |
| Stent thrombosis | 1 (1.2) | 1 (0.7) | >0.99 |
| Target vessel revascularisation | 3 (3.6) | 4 (3.0) | >0.99 |
| Percutaneous coronary intervention | 1 (1.2) | 3 (2.2) | >0.99 |
| Coronary artery by-pass graft surgery | 2 (2.4) | 1 (0.7) | 0.56 |
| Heart failure hospitalisation | 1 (1.2) | 5 (3.7) | 0.41 |
| Data are presented as n (%) or mean (± SD). ^*^Peak troponin level was divided by the upper reference limit of the corresponding assay. ^†^Performed on pre-discharge ECGs acquired at a median time of 51 hours from fibrinolysis (IQR, 24-82). ^‡^ST-segment recovery is expressed as a per cent change from baseline in the lead with maximum ST-segment elevation and presented as n (per cent of ECGs analysed). ECGs were acquired post-fibrinolysis at a median time of 75 minutes (IQR, 60-96) and post-PCI at a median time of 32 minutes (IQR, 14-49). ACUITY = Acute Catheterization and Urgent Intervention Triage Strategy; ECG = electrocardiogram; TIMI = Thrombolysis in Myocardial Infarction; URL = upper reference limit. | | | |
