# Supplementary material for: Reperfusion After Fibrinolytic Therapy (RAFT): An open-label, multi-centre, randomised controlled trial of bivalirudin versus heparin in rescue percutaneous coronary intervention
Source: PLoS One. 2021 Oct 26;16(10):e0259148. doi: 10.1371/journal.pone.0259148 (PMC8547635; doi:10.1371/journal.pone.0259148)
Supplement: S2 File — RAFT trial protocol. (PDF) [file pone.0259148.s003.pdf]

Reperfusion after fibrinolytic therapy (RAFT)

Investigators Protocol

Final Protocol Version: September 2009
Amendment 1 Version: January 2012
Amendment 2 Version: August 2014

Names and Addresses of:

PRINCIPAL INVESTIGATOR

Professor John French
Department of Cardiology
Liverpool Hospital
Elizabeth Street
Liverpool NSW 2170
Australia
Tel: (61) 2 8738-3069 (Office)
Tel: (61) 2 8738-3495 (Sec)
(61) 2 8738-3011_25677 (Pager)
Fax (61) 2 9828-3341
j.french@unsw.edu.au

Project MANAGEMENT

Adelaide Clinical Research
SAHMRI
North Terrace Adelaide SA 5000
Australia
Tel: (61)8 81284504

DATA MANAGEMENT

HSR SAHMRI
SAHMRI
North Terrace Adelaide SA 5000
Australia
Tel :1300 725 272
HSR@sahmri.com

PROTOCOL AGREEMENT FORM

I, Principal Investigator, have examined this amended protocol for the RAFT study

Entitled: “RAFT” Trial

Date: ____________________________

And I have fully discussed the objectives of this clinical trial and the contents of this protocol with the Chief Investigator representative(s).

I agree to conduct the clinical trial according to this protocol and to comply with its requirements, subject to ethical considerations and acknowledge that I am responsible for the overall study conduct.

I agree to keep confidential the content of the protocol, not to disclose it to any third party and to use it only for the purpose of conducting this trial.

I understand that, should the decision be made by the Chief Investigator to terminate prematurely or suspend the clinical trial at any time for whatever reasons; such decision will be communicated to me in writing. Conversely, should I decide to withdraw from execution of the clinical trial I will communicate immediately such decision in writing to the Chief Investigator or their representative.

**Principal Investigator**	**Chief Investigator (or representative)**
NAME:	NAME:
SIGNATURE:	SIGNATURE:
DATE: ________________	DATE: _________________

TABLE OF CONTENTS

1. ABBREVIATIONS AND DEFINITIONS....................4
2. SYNOPSIS.................... 8
3. Study Flow Chart.................... 10
4. SUMMARY.................... 15
5. INTRODUCTION AND RATIONALE.................... 13
5.1 Background....................13
6. AIMS AND OBJECTIVES.................... 15
6.1 Primary Endpoints....................15
6.2 Secondary Endpoints....................15
7 STUDY DESIGN.................... 16
8 STUDY POPULATION.................... 16
8.1 Patient Information and Consent.................... 16
8.2 Number of patients planned....................16
8.3 Inclusion Criteria....................16
8.4 Exclusion Criteria....................17
9 CONCOMITANT THERAPY.................... 17
10 OBSERVATIONS.................... 18
10.1 Measurements and Investigations....................18
10.2 Investigational Plan....................18
10.3 Laboratory....................18
11 Endpoint Definitions.................... 19
12 SUBSTUDIES.................... 21
13 STATISTICS.................... 21
13.1 Statistical Design/Model....................21
14 MONITORING....................19
15 ADVERSE DRUG REACTION REPORTING.................... 20
16 ETHICS....................21
17 REFERENCES.................... 25

1 ABBREVIATIONS AND DEFINITIONS

ACT	Activated Clotting Time
ADR	Adverse Drug Reaction
AE	Adverse Event
AMI	Acute Myocardial Infarction
AUC	Area under curve
B-blockers	Beta blocker drugs
BP	Blood Pressure
CABG	Coronary Artery Bypass Graft
CAPTIM Study	Comparison of primary angioplasty & Pre hospital fibrinolysis in Acute Myocardial Infarction
CARESS Study	Dual antiplatelet therapy with clopidogrel and aspirin in symptomatic carotid stenosis evaluated using doppler embolic signal detection: the Clopidogrel and Aspirin for Reduction of Emboli in Symptomatic Carotid Stenosis (CARESS) trial.
CCL	Cardiac Catherisation Laboratory
CK	Creatine Kinase
CKMB	Creatine Kinase Muscle Brain
CT	Computed Tomography Scan
cTFC	corrected TIMI Frame Count
CVA	Cerebrovascular Accident
DAN AMI -2 Study	Danish Trial in Acute Myocardial Infarction-2
eCRF	Electronic Case Report Form
ECG	Electrocardiogram
FAST MI Registry	The French **registries** of Acute ST-elevation and non-ST-elevation Myocardial Infarction
FU	Follow Up
g/dl	grams per decilitre
GI Bleed	Gastric intestinal bleed
GU Bleed	Genitourinary bleeding
GPIIb/IIIb	Glycoprotein Platelett Inhibitor
HB	Haemoglobin
HREC	Human Research Ethics Committee
HORIZONS-AMI Study	Harmonizing Outcomes with RevasculariZation & Stents in Acute Myocardial Infarction
Hr	Hour
hs	High sensitivity
ICH	International Conference on Harmonisation
IEC's	Independent Ethics Committee

INR	International Normalized Ratio
IRA	Infarct Related Artery
IRB	Institutional Review Board
IV	Intravenous
Killip Class	A system used in individuals with an acute myocardial infarction (heart attack), in order to risk stratify them. Individuals with a low Killip class are less likely to die within the first 30 days after their myocardial infarction than individuals with a high Killip class.[1]
LATE Study	The late Assessment of Thrombolytic Efficacy Trial
LBBB	Left bundle Branch Block
LMWH	Low Molecular Weight Heparin
MERLIN Study	The Middlesborough Early Revascularization to Limit Infarction
mg	milligram
MI	Myocardial Infarction
mm	millimetre
mmHg	Millimetres of Mercury
MRI	Magnetic Resonance Imaging Scan
ms	millisecond
OAT Study	Occluded Artery Trial
P2y12	Regulator in blood clotting
PCI	Percutaneous Coronary Intervention
PI	Principal Investigator
PO	Per Oral
QRS Complex	is a name for the combination of three of the graphical deflections seen on a typical electrocardiogram (ECG). It is usually the central and most visually obvious part of the tracing. It corresponds to the depolarization of the right and left ventricles of the human heart
REACT Study	Rescue Angioplasty Versus Conservative Treatment
SADR	Serious Adverse Drug Reaction
SAE	Serious Adverse Event
SAER	Serious Adverse Event Reporting
ST Segment	line from the end of the QRS complex to beginning of T wave
STEMI	ST Elevation Myocardial Infarction
STREAM	Strategic Reperfusion early After Myocardial Infarction
TIA	Transient Ischaemic Attack
TIMI	Thrombolysis in Myocardial Infarction
TnT T	Troponin Cardiac Marker in Ischemic Heart Disease

TPMG	TIMI Myocardial Perfusion Grade
TRANSFER AMI Study	Trial of Routine ANgioplasty and Stenting after Fibrinolysis to Enhance Reperfusion in Acute Myocardial Infarction - The TRANSFER-AMI trial
U/kg	units per kilogram
UFH	Unfractionated Heparin
ULR	Upper Limits of Reference Range

.

2 SYNOPSIS

**Title**	RAFT - Reperfusion After Fibrinolytic Therapy<br>Trial registry: www.anzctr.org.au; identifier: ACTRN12610000152022.
**Trial Location**	Australia, New Zealand and India
**Aim**	To show that the use of bivalirudin has a lower rate of ACUITY bleeding and is equally efficacious compared to heparin (UFH/ LMWH) +/- glycoprotein IIb/IIIa (GPIIb/IIIa) inhibitors as assessed by infarct size (cardiac marker area under the curve) in the setting of pharmaco-invasive PCI
**Study Design & Duration**	Investigator-initiated, multicentre, randomised, open-label, parallel group clinical trial.<br>Duration: 5 years<br>The trial was funded by an unrestricted educational research grant from The Medicines Company (New Jersey, USA) with no access to the trial data and no role in the trial design, conduct, analysis and reporting of the trial.
**Population**	<u>Inclusion Criteria</u><br>1. Age > 18 years.<br>2. Patients with STEMI presenting within 12 hours of onset of symptoms<br>3. Presence of ST elevation in 2 contiguous leads of ≥ 2mm for leads V1-V3, or ≥ 1mm for other leads, or for posterior MI ≥ 1mm ST depression for leads V2-V3.<br>4. Administration of fibrinolytic therapy<br>5. Provision of informed consent.<br>6. The decision to perform PCI (rescue or pharmaco-invasive) within 24hrs of STEMI<br><u>Exclusion Criteria</u><br>1. Hypertension with blood pressure persistently >180/110mmHg.<br>2. Significant bleeding disorder or recent major bleeding within 3 months, including on Warfarin therapy (INR >2).<br>3. Known history of intracranial haemorrhage or trauma.<br>4. Known history of ischaemic stroke or recurrent TIA.<br>5. Known hypersensitivity or contraindication to bivalirudin, heparin, GPI and any other medications that may be used in the study.<br>6. Previous reactions to contrast agents.<br>7. Severe renal impairment (creatinine clearance <30ml/min or creatinine ≥ 250μmol/L).<br>8. Unavailability for follow up.<br>9. Female subjects of childbearing potential.
**Drug Dosing**	All drugs (Bivalirudin, Heparin or Enoxaparin +/- GPIIb/IIIa antagonists) will be administered as per product information.
**Main Evaluation Criteria**	The primary safety endpoint will be ACUITY bleeding (major, moderate and minor).<br>The primary efficacy endpoint will be infarct size assessed by the area under the curve (AUC) of CK-MB levels.
**Main Data Collection**	Data will be collected on Electronic Case Report Forms (eCRF)<br>Data on Participants (from hospitalization to discharge)<br>○ *Demography*<br>○ *Medical history*<br>○ *Date and time of symptom onset*<br>○ *Date and time of admission (Referral and PCI Hospital)*<br>○ *Laboratory analysis*

	○ *Diagnostic ECG*<br>○ *In-patient therapies*<br>○ *In-patient events*<br>○ *In-patient procedure*<br>○ *Medications during hospitalisation and at discharge*<br>○ *Reperfusion Score*<br>Data on Participants (30 and 90 days) by visit or phone contact, General Practitioner contact, next of kin contact and/or hospital admission database<br>○ *Outcome*<br>○ *Medications taken*
**Statistical Methodology**	Sample size calculation<br>A total sample size of 410 patients has 80% power ($p \leq 0.05$) of determining a 40% reduction in the ACUITY bleeding endpoint in the bivalirudin arm compared to the UFH/LMWH -/+ GPIIb/IIIa therapy arm.<br>An interim analysis will be performed on the first 280 patients enrolled in the study and an adaptive sample size re-estimation design will be employed at that time.
**Timelines**	Aust/NZ FPI Aug 2010<br>India FPI India =16 sites<br>Planned interim analysis @ n=280 Dec 2015

3 STUDY FLOW CHART

Pre-transfer			Follow-up									
	Pre lytic Baseline*	Post lytic 60-90 mins*	Inclusion	Pre PCI	1-5.9 hours	6-15.9 hours	16-29.9 hours	30-59.9 hours	60-96 hours	Pre-discharge	30 Day FU	90 Day FU
**Inclusion/Exclusion Criteria**			X									
**Consent**			X									
**Randomisation**			X									
**Diagnostic ECG**	X	X		X	X (0-90mins)		X			X	X[c]	
**Cardiac markers (CKMB, TnT)**	X[a]	X[a]		X [a]	X	X	X	X	X[e]			
**Electrolytes including Glucose**	X			X[b]								
**Haematology**	X	X		X			X			X[d]		
**Concomitant Medications**										X	X	X
**History & Physical Examination**			X									
**Outcomes**										X	X	X

X required; X[a] At least one of these samples to be taken; X[b] Only necessary if baseline not done; X[C] Can be sourced from GP; X[d] Please record haematology results with the lowest Hb post rescue (no extra blood sample to be collected)

4 SUMMARY

Fibrinolytic therapies will continue to be administered, especially in the pre-hospital or community hospital settings, to patients with ST-segment elevation myocardial infarction (STEMI) as the ability to perform timely 24-hour primary percutaneous coronary intervention (PCI) has major resource and logistic implications[1]. Thus, in circumstances where it is not possible to routinely achieve guideline-recommended times from first medical contact to initial device use at PCI, fibrinolytic therapy (in the absence of contra-indications) should be administered. A pharmaco-invasive strategy involving early transfer for angiography is supported by TRANSFER-AMI, CARESS, STREAM, the CAPTIM-WEST combined data and FAST-MI. This has resulted in recent guidelines recommending patients who successfully achieve pharmacologic reperfusion undergo angiography, and if indicated PCI, within 3-24 hours.

Patients who are considered to be at low likelihood of having achieved reperfusion after fibrinolysis should be transferred for emergency angiography, and if indicated rescue PCI. These recommendations are largely based on a meta-analysis of 5 trials of outcomes after Rescue Angiogrplasty[3]. Two trials from the UK provided the majority of the patients, MERLIN and REACT. In REACT, patients undergoing rescue PCI had better clinical outcomes at 6 months than those managed either with conservative therapy or re-administration of fibrinolytic therapy[2]. However, when these trials were conducted different interventional techniques and therapies were used. Which adjunctive pharmacologic therapies should be used in the pharmaco-invasive strategy remains unclear. In particular, clinicians have been concerned about the risk of bleeding associated with the routine use of glycoprotein IIb/IIIa antagonists in patients who have received full doses of fibrinolytic therapies, aspirin, clopidogrel and adjunctive intravenous unfractionated heparin (UFH), or enoxaparin. Patients with STEMI undergoing early PCI following fibrinolytic therapy are at a higher bleeding risk and in the era prior to routine stenting when longer activated clotting time (ACT) were used, patients receiving bivalirudin had reduced bleeding compared to UFH. Also in patients undergoing PCI for either stable angina or non-ST elevation acute coronary syndromes, randomisation to receive bivalirudin compared to standard regimens of intravenous heparin and a glycoprotein IIb/IIIa antagonist (abciximab) was associated with similar efficacy in prevention of ischemic events but reduced bleeding[4-6]. Furthermore, patients undergoing primary PCI and receiving bivalirudin, compared to those receiving UFH and a glycoprotein IIb/IIIa antagonist, had both reduced bleeding and mortality[7]. Whether bivalirudin reduces bleeding compared to heparin and a glycoprotein IIb/IIIa antagonist, clinician commenced, while maintaining efficacy during a pharmaco-invasive strategy performed with current interventional techniques, including thienopyridine pre-treatment, should be assessed.

In this study, patients transferred for a pharmaco-invasive strategy will be randomised in a 1:1 fashion to receive either bivalirudin or unfractionated heparin (UFH) or enoxaparin with or without glycoprotein IIb/IIIa (GPIIb/IIIa) antagonists at the interventionalist's discretion. Patients administered heparin should remain on therapy until arrival at the Cardiac Catheterisation Laboratory (CCL). For patients who are already on either Heparin and/or a short acting GPIIb/IIIa antagonists (patients on *abciximab* are excluded) at the time of randomisation, those randomised to receive bivalirudin should have prior therapy ceased immediately. Recruitment will be stratified according to the radial or femoral procedural approach to PCI.

4.1 Outcomes

The **primary safety endpoint** will be ACUITY bleeding. It is hypothesised that randomisation to receive bivalirudin will result in a lower rate of ACUITY bleeding (major and minor), compared to heparin and a GPIIb/IIIa antagonist. Secondary safety endpoints will be the change in haemoglobin level, corrected for transfusions, and rates of TIMI Major and Minor bleeding.

The **primary efficacy endpoint** will be infarct size assessed as the area under the curve (AUC) of CKMB levels. The hypothesis is the non-inferiority of the bivalirudin alone strategy compared to heparin +/- GPIIb/IIIa antagonists. Secondary endpoints will include the infarct size determined by the Selvester QRS score on electrocardiograms, and serial high sensitivity (hs) Troponin T levels.

Angiographic parameters to be measured include infarct artery TIMI flow grade, corrected TIMI frame count (cTFC), and TIMI myocardial perfusion grade (TMPG). Tertiary endpoints include clinical outcomes of death, recurrent myocardial infarction, stroke, urgent target vessel revascularization, stent thrombosis, highest Killip class, and re-hospitalisation for heart failure over 90 days.

SUMMARY OF PROTOCOL DIAGRAM – RAFT

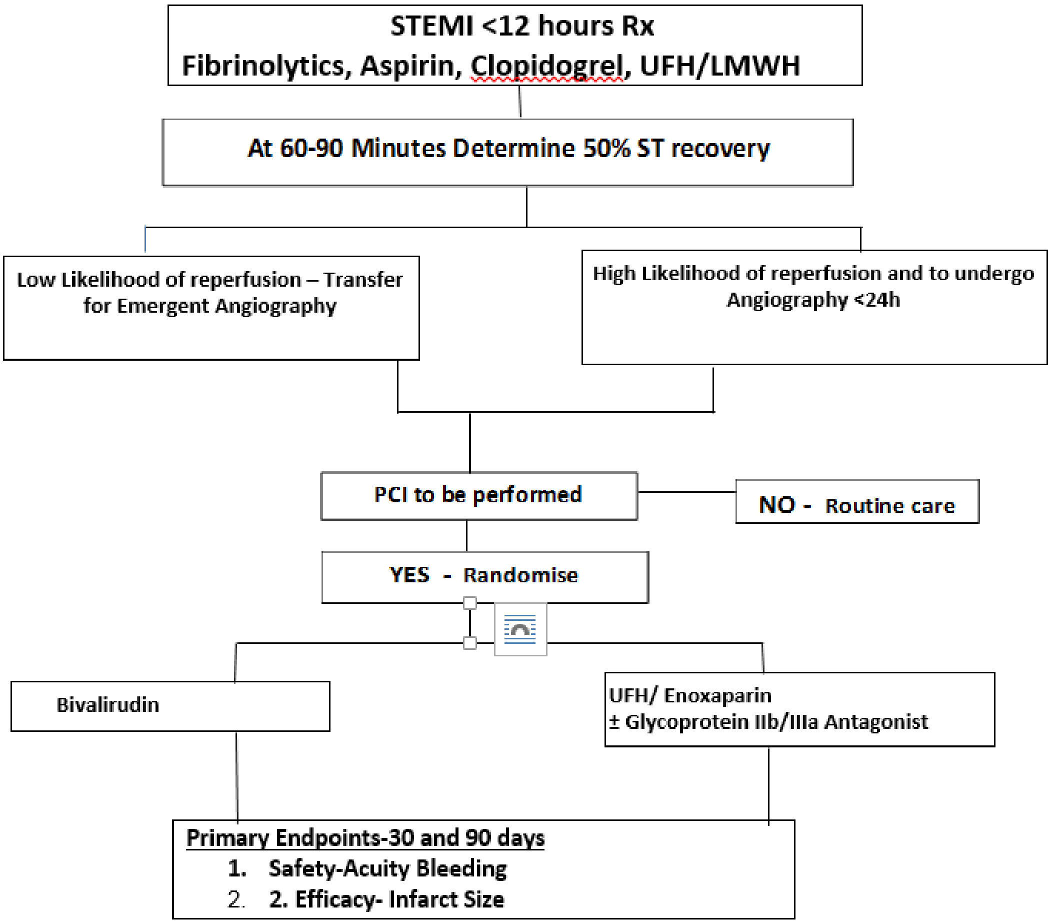

INTRODUCTION AND RATIONALE

5.1 Background

ST Elevation Myocardial Infarction

Pharmacologic reperfusion with intravenous fibrinolytic and anti-thrombotic agents has been shown unequivocally to reduce mortality in patients with STEMI, including those with presumed new onset left bundle branch block (LBBB), presenting within 12 hours of symptom-onset[8]. While , meta-analysis revealed lower mortality (and re-infarction) rates following mechanical reperfusion with primary PCI compared to fibrinolytic therapy there were variable rates of angiography and PCI during the initial hospitalization[9]. These data are derived from patients treated in various health care settings including community hospitals, regional tertiary centres and in ambulances; various pharmacologic therapies and interventional techniques were used. The potential advantages in administration of pre-hospital fibrinolysis especially in those presenting early (within 2 hours of symptom-onset) [11-13]have been reported in Registry Studies.

While the emergent transfer of all fibrinolytic treated patients with STEMI require considerable resources including afterhours CCL staffing and ambulance services, those who fail to reperfuse or have cardiogenic shock have been shown to benefit from emergency revascularization, if necessary, after immediate inter-hospital transfer. These patients have a life-threatening medical emergency somewhat analogous to major trauma, emergency angiography is recommended and rescue PCI if indicated. Angiography is now guideline-recommended 3-24 hrs post 'lysis in for STEMI patients without contra-indications [STEMI guidelines 2012 ESC 2013 AHA]. In Australia, 85-90% of patients undergo angiography at some time during their initial hospitalization[14]. Current evidence does not support immediate angiography and PCI after successful 'lysis' known as facilitated PCI[15] though data from the TRANSFER AMI trial[16] favours angiography after ~3 hours in all post 'lytic' patients, not just patients who, at 60-90 mins after lytic administration, failed to achieve 50% ST recovery[17-19]. Systems of care have been recommended to improve time to treatment. Data from France and some US regions (such as Minnesota and North Carolina) support a pharmaco-invasive approach to STEMI care. [20-23] Whether full or half dose fibrinolytics should be used remains contentious, though the STREAM trial supports 1/2dose lysis in the elderly undergoing a Pharmaco-invasive strategy

A Pharmaco-invasive PCI strategy involving emergent angiography and rescue PCI in participants who fail to achieve 50% ST recovery at 60-90 min and early angiography PCI indicated those who reperfuse is attractive [Heart 2009 Vanderwal NEJM 2013]. This approach was compared to primary PCI in patients presenting at <3hrs who had a >60 min anticipated delay time in the STREAM trial. This trial had a trend towards improved outcomes in the pharmaco-invasive group like CAPTIM there was less shock in those undergoing the plasma invasive strategy. Whether a predominantly radial approach which is used in contemporary primary PCI affects recommendations regarding bleeding associated with post lytic PCI is not clear.

Data from the STREAM study reports major bleeding rates of 9.6% and 5.1% for rescue and Pharmacoinvasive PCI respectively (Welch RAJ on line 2014) The rates of both rescue PCI to achieve reperfusion when fibrinolytic therapy has failed and early non-emergent percutaneous or surgical revascularisation following fibrinolytic therapy, have varied markedly in a meta-analysis of trials examining strategies of transfer for primary PCI compared to (local) administration of fibrinolysis[9]. These trials recruited patients in a variety of clinical settings and healthcare systems, ranging from community hospitals to regional tertiary centres, and used various drug therapies and interventional techniques[9]. For example, DANAMI-2[26] reported a very low rate (2.6%) of rescue PCI which significantly contributed to the meta-analysis which does not reflect contemporary practice, whereas not included, was the CAPTIM trial which reported very high rates of rescue PCI and ad-hoc PCI[11]

during initial hospitalisation. CAPTIM five year follow up has shown improved late survival among those randomised to pre-hospital fibrinolysis at <2hours (compared to primary PCI) similar results to those subsequently reported from the FAST-MI registry[12]. Data of consecutive patients over the last decade undergoing rescue PCI at Liverpool Hospital, Sydney Australia, (90% after tenecteplase) and with about 80% glycoprotein IIb/IIIa antagonist use mainly 'upstream showed 30-day mortality and 3.2% in those patients without cardiogenic shock[22 (Shugman/et al AHJ 2012)], similar to that reported for primary PCI. Bleeding rates were 25% for any bleeding, 6.6% for TIMI Major and, the transfusion rate was 10%.

The utility of rescue PCI following fibrinolysis for STEMI patients was debated during the 1990s but received evidential support from the meta-analysis performed by Ellis and co-workers in 2000[24] which showed that patients with TIMI 0-1 flow had better outcomes after rescue PCI than with conservative therapy. Another meta-analysis in 2007 which included 900 patients, largely reflecting results from 2 randomised trials conducted in the United Kingdom as well as 3 small trials, reported improved outcomes and a trend towards mortality advantage for rescue PCI[3]. The **M**iddlesbrough **E**arly **R**evascularisation to **L**imit **I**nfarctio**N** (MERLIN) trial randomised 307 patients who failed to achieve50% ST recovery at 60 minutes following fibrinolytic therapy (60% streptokinase), to either rescue PCI or conservative treatment[25], repeat administration of fibrinolytic therapy was discouraged. There was no difference in the outcomes of mortality or left ventricular function at 30 days, though there was improved event-free survival in the rescue PCI arm, largely due to a decrease in subsequent revascularisation (6.5% vs 20.1%, p<0.01). The REACT study of 427 patients, which used <50% ST recovery at 90 minutes as an entry criterion, demonstrated a 50% reduction over 6 months in the composite of mortality, stroke, severe heart failure and recurrent myocardial infarction in patients undergoing rescue PCI compared to those randomised to either fibrinolytic re-administration or conservative therapy[2]. A post-hoc analysis showed reduced 6 month mortality in the Rescue PCI arm compared to the other 2 arms combined[3].

In making triage decisions for rescue PCI, time is of the essence and the non-invasive evaluation of reperfusion needs to be quickly performed at the bedside. Early studies using intracoronary streptokinase in patients with STEMI described four features associated with recanalisation of the infarct-related artery (IRA), relief of chest pain, the development of reperfusion arrhythmias, resolution of ST elevation (known as ST recovery) on the electrocardiogram (ECG), and the rapid release of biochemical markers[27,28]. While the prompt resolution of chest pain should be a goal in all patients, complete resolution of pain has been shown to occur in a minority of patients with patent arteries[29] and this feature has not been shown to be a consistent guide to the likelihood of reperfusion. Neither is the presence of accelerated idioventricular rhythm sensitive enough to be of value in assisting triage decisions, despite this classic reperfusion arrhythmia being specific for patency of the IRA[30] Patients with a 50% ST recovery (at 60-90 min) and with persistent (or early recurrent) ischemic chest pain are indicated for rescue PCI[15, 18].

The degree of ST recovery following fibrinolytic therapy is associated with patency and flow in the IRA; >70% ST resolution is associated with a 90-95% probability of achieving a patent IRA[31,32]. Single lead ST-segment measurement has been found to be as good a predictor as multi-lead measurements[33], despite being less complex and easier to calculate at the bedside. However, ST resolution is an imperfect discriminator between TIMI grade 2 and 3 flows, with up to 50% of patients with persistent ST elevation having a patent IRA at the time of angiography[30,34]. Thus the lack of ST resolution can indicate failure of perfusion at a myocyte/microvascular level[35], and in these patients persistent ST elevation is associated with more extensive myocardial damage and a higher long-term mortality rate[32]. While a reperfusion score can predict the likelihood of failed pharmacological reperfusion (failure to achieve TIMI-3 flow at 90 minutes) including ST recovery in the lead with maximal ST elevation and Troponin T levels at baseline and at 60 minutes after fibrinolytic therapy[8], this has not been validated with point of care troponin assays to facilitate 'real-time' use.

Antithrombotic and glycoprotein IIb/IIIa inhibitor therapies

In patients with STEMI treated with fibrinolytic therapy who underwent early PCI, the bleeding rates with bivalirudin were lower in an earlier era among fibrinolytic-treated patients, compared to unfractionated heparin[38]. In separate studies, patients undergoing angioplasty for either stable angina or non-ST elevation acute coronary syndromes, who were randomised to receive standard boluses (and infusions) of bivalirudin, compared to intravenous heparin and glycoprotein IIb/IIIa antagonists (abciximab), had similar rates of ischaemic events, but reduced bleeding[5,6]. Interestingly the HORIZONS-AMI trial[7] showed a mortality reduction in STEMI patients planned to undergo primary PCI associated with randomisation to bivalirudin compared to unfractionated heparin and a glycoprotein IIb/IIIa inhibitor (mainly abciximab or eptifibatide) which remained significant at 3 years follow up. In this study, there was a 5.5 fold mortality risk associated with early stent thrombosis, whereas the mortality risk for major bleeding was 7.5 fold, emphasizing the importance of bleeding as both an adverse clinical outcome and as a trial endpoint.

The question of which pharmacologic strategies should be used in the setting of rescue and early Pharmacoinvasive remains unclear and some clinicians at referral centres, concerned about transfer delays and anxious to provide some treatment, initiate glycoprotein IIb/IIIa therapies[22]. Other clinicians are concerned about the bleeding risks associated with the use of glycoprotein IIb/IIIa antagonists in patients who have received full doses of fibrinolytic therapies, aspirin, thienopyridines and UFH/LMWH. While prior to, and during, primary PCI platelet inhibition following loading with clopidogrel 300 mg, the most frequently used dose in HORIZONS-AMI, was likely to be suboptimal given its requirement to be metabolised from a pro-drug, this agent may contribute to bleeding risks post-PCI. Newer agent thienopyridines have not been studied in the setting of rescue PCI. In this setting, whether bivalirudin has a better safety profile than UFH/LMWH +/- GPIIb/IIIa antagonist, while maintaining efficacy, this requires clarification.

6 AIMS AND OBJECTIVES

This study to be conducted in patients with STEMI who are treated with fibrinolytics, aims to test the following hypothesis: that the use of bivalirudin has a lower rate of ACUITY bleeding and is equally efficacious compared to heparin (UFH/ LMWH) +/- GPIIb/IIIa inhibitor as assessed by infarct size (CKMB cardiac marker area under the curve) in the setting of early post lysis PCI (at <24hrs) including rescue PCI for failed reperfusion.

6.1 Primary Endpoints

The primary endpoint of this study is the safety endpoint of ACUITY (major, moderate and minor) bleeding at 90 days. A superiority comparison of bivalirudin to the 'control' arm UHF/LMWH +/- GPIIb/IIIa inhibitor therapy will be performed.

The primary efficacy outcome will be infarct size assessed as the area under the curve (AUC) of CKMB levels. Non-inferiority of the bivalirudin alone strategy compared with UHF/LMWH +/- a GPIIb/IIIa antagonist will be examined.

6.2 Other Endpoints

The secondary safety endpoint will be the change in haemoglobin level between the level immediately prior to angiography and the level the next day. Rates of transfusion will be captured, and haemoglobin drop corrected for units of red blood cells transfused.

Secondary efficacy endpoints will include the final infarct size (hence the need for a pre-discharge ECG) determined by electrocardiographic Selvester QRS score assessed as continuous variable, and AUC of hs Troponin T. ST recovery will also be determined.

Angiographic characteristics of pre- and post- PCI infarct artery TIMI flow grade, TIMI myocardial perfusion grade (TMPG) and corrected TIMI frame count (cTFC) and if measured left ventricular function indices will be assessed. Tertiary endpoints include clinical outcomes of death, recurrent myocardial infarction, stroke, urgent target vessel revascularization, stent thrombosis, highest Killip class, and re-hospitalisation for heart failure over 90 days.

7 STUDY DESIGN

See protocol flow diagram (Section 3. Summary)

Patients referred for angiography <24h post- fibrinolysis, who are to undergo PCI including rescue PCI, will be randomised in a 1:1 ratio (in permuted blocks using a concealed and centralised electronic system) to receive either bivalirudin or UHF/LMWH +/- glycoprotein IIb/IIIa inhibitors at the operator's discretion. It should be noted that patients on upstream small molecule GP IIb/IIIa inhibitors (tirofiban or eptifibatide) are eligible if the investigator agrees to immediately discontinue such an agent if randomisation to receive to bivalirudin occurs. Recruitment will be stratified by femoral or radial procedural approach. All patients with STEMI treated with fibrinolytic therapy, aspirin, UFH/LMWH, and a thienopyridine, undergoing angiography at <24h are potentially eligible. Patients (see Section 4) will have baseline blood samples, cardiac markers (point-of-care where available) and an ECG to measure ST elevation. At 60-90 minutes repeat ECG and blood tests will be performed unless there is a clinical indication (e.g. cardiogenic shock) to undertake angiography emergently. Patients who are unlikely to have reperfused based on <50% ST recovery on an ECG at 60-90 minutes and who are being transferred for rescue PCI, are eligible.

8 STUDY POPULATION

All patients with STEMI presenting <12 hours after symptom onset, who are treated with fibrinolytics and, who are to undergo either rescue PCI or pharmaco-invasive PCI at <24hrs

8.1 Patient Information and Consent

The Patient Information/Informed Consent form will be reviewed and approved by the IRB/HREC for each of the sites. Inclusion in the study will occur only if the patient gives written informed consent. Each study patient will be informed both verbally and in writing about the nature of the study, the anticipated risks and benefits, the discomforts to which the patient may be exposed, and their right to discontinue participation at any time of their own free will, and about the compensation in case of trial related injury during clinical trial. Informed consent of subjects participating in this study should include the consent for access to subject's source documents.

8.2 Number of patients planned

It was originally planned that 410 patients with STEMI treated initially with fibrinolytic therapies undergoing Rescue PCI would be randomised. Study power is based on a 40% reduction (18% v 30%) in the bivalirudin arm of the ACUITY (major and minor) bleeding endpoint (α=0.80, β=0.05).

Using an adaptive statistical design, to determine the actual event rate and any requirement to change the total number randomised, an interim analysis will be performed after the recruitment of 280 patients undergoing either Rescue or Pharmaco-invasive PCI < 24hrs (see Section 13).

8.3 Inclusion Criteria

1. Age > 18 years.

2. Patients with STEMI presenting <12 hours from symptom
3. Presence of ST elevation in 2 contiguous leads of ≥ 2mm for leads V1-V3, or ≥ 1mm for other leads, or for posterior MI ≥ 1mm ST depression for leads V2-V3.
4. Administration of fibrinolytic therapy.
5. Provision of informed consent.
6. The patient has undergone angiography <24h post-STEMI and he/she is about to undergo PCI, either rescue or pharmaco-invasive.

8.4 Exclusion Criteria

1. Hypertension with blood pressure persistently >180/110mmHg.
2. Significant bleeding disorder or recent major bleeding within 3 months, including on Warfarin therapy (INR >2).
3. Known history of intracranial haemorrhage or trauma.
4. Known history of ischaemic stroke or recurrent TIA.
5. Known hypersensitivity or contraindication to bivalirudin, heparin, GPI and any other medications that may be used in the study.
6. Previous reactions to contrast agents.
7. Severe renal impairment creatinine clearance <30ml/min or creatinine ≥ 250µmol/L.
8. Unavailability for follow up.
9. Female subjects of childbearing potential.

9 CONCOMITANT THERAPY

Eligible patients who provide written informed consent and are treated with fibrinolytic therapy including enoxaparin or intravenous heparin 60 U/kg bolus (max 4000 U) + 12U/kg infusion (max 1000 U/hr) must have these drugs continued until angiography. Patients started on upstream tirofiban or eptifibatide are eligible if, on randomisation to receive bivalirudin, the interventional cardiologist is agreeable to immediately discontinue glycoprotein IIb/IIIa inhibitor therapy. Also, evidence-based therapies including aspirin, statin, β-blockers and angiotensin converting enzyme inhibitors or blockers are recommended.

Anti-platelet Agents

All patients will be given, 150 – 325 mg PO of non-enteric-coated aspirin immediately upon admission / study entry and then 75 – 325 mg PO (coated or uncoated) once daily for at least 90 days. The only exception to this regimen is for patients who have taken aspirin within the previous 12 hours; these patients will start aspirin the next day. All patients should also receive a clopidogrel loading dose of a minimum of 300 mg PO (consider only 75mg if age > 75 years), and a maintenance dose of at least 75 mg PO daily. Standard loading and maintenance doses for other P2Y12 antagonists approved for post-‘lytic PCI are permissible as alternatives to clopidogrel.

Glycoprotein IIb / IIIa antagonists

In the bivalirudin arm during the PCI procedure, if recurrent clot formation or other events such as side branch occlusion endanger the procedural success and clinical outcome after other strategies (e.g. thrombus aspirations) have been undertaken. Glycoprotein IIb/IIIa antagonists may be used at the discretion of the operator/investigator in a “bail-out” circumstance. Note patients who have received small molecule glycoprotein IIb/IIIa antagonists are eligible, if the investigator considers it appropriate that the infusion is immediately stopped if the patient is randomised to receive bivalirudin.

Drugs will be available for the patient from the hospital supply.

10 OBSERVATIONS

10.1 Measurements and Investigations (see study flow chart)

- Medical history and physical examination
- Electrocardiogram (ECG)
    - Pre lytic from referral hospital
    - 60-90 minutes post lytic from referral hospital (where available)
    - Pre PCI (0-30 minutes)
    - 0-90 minutes post PCI
    - 18-24 hours post PCI
    - Pre discharge
- Biochemical markers (CK, CKMB) and Troponin T or I at
    - Pre lytic from referral hospital (important). Point of care Troponin T or I (where available)
    - 60-90 minutes post lytic from referral hospital (where available)
    - 0-30 minutes pre PCI
    - 1-5.9 hours post PCI
    - 6-15.9 hours post PCI
    - 16-29.9 hours post PCI
    - 30-59.9 hours post PCI
    - 60-96 hours post PCI (pre discharge)
- Haematology (Hb, haematocrit, platelets, white cell count)
    - Pre lytic
    - Pre PCI (0-30 minutes)
    - Post PCI (6-24 hours, Day 1)
    - Post PCI to discharge lowest Hb (no extra sample required)
- Electrolytes and Creatinine at Baseline
- Killip Class assessment (baseline and worst)
- Arterial Access Route

10.2 Investigational Plan (Section 3)

- Pre-PCI period
- In-hospital period (pre-and post-PCI)
- Concomitant medication
- Cardiac catheterization and revascularization (PCI)
- Clinical assessment of efficacy and safety
- Hospital discharge
- 30 day follow up visit or phone call and 90 day follow-up call

10.3 Core Laboratory Measurements (Section 3)

- CKMB and hs troponin T core lab
- ECG core lab
- Angiographic core lab

Cardiac marker analyses

Infarct size assessed by CKMB AUC is the primary efficacy endpoint in the study. Serial samples will be collected within the protocol specified windows. Serial blood samples will be obtained during the initial 96 hours of the index hospitalization for determination of cardiac biomarkers. Curve fitting will be performed according to accepted methodologies to generate CKMB and cardiac troponin T or I estimated peak and AUC. Assays will include:

- CKMB AUC and estimated peak
- Time to estimated peak CKMB
- Cardiac hs troponin T or I AUC and estimated peak

ECG analyses

ST recovery measurement on the baseline and post-thrombolytic ECGs will be as follows: (1) as the changes in the sum of the voltages in all leads with ≥1 mm ST deviation, and (2) as the changes in voltage in the single lead with maximum ST elevation. With the TP segment as the iso-electric line, ST elevation or ST deviation (i.e., ST elevation and ST depression, if present) are measured to the nearest 0.5mm in all leads at 60 milliseconds after the J point. Qualifying baseline ECGs will be recorded ≤120 minutes before or ≤6 minutes after institution of thrombolytic therapy.

ECG's will be deemed technically unsuitable if they are incomplete or of poor quality, or if there were conduction abnormalities, ventricular hypertrophy, or a ventricular or paced rhythm. The sum of the voltages in all leads with ST deviation on the baseline ECG and the voltage in the lead with maximum ST elevation will be recorded. ECG measurements will be made manually with callipers by 2 experienced analysts, who will be blinded to treatment allocation and clinical outcome. QRS scoring will be performed as previously described[14].

Angiographic Analyses

All angiographic analysis will be performed in a core lab off-line by 2 experienced readers who are unaware of treatment allocation. The name, identification, and date of angiography will be removed / covered to ensure the reader has no access to any clinical data. The readers will record TIMI flow grades, frame counts and blush grade of the infarct artery at 2 time points: on angiography prior to PCI and the final imaging post PCI. Other parameters include the percentage diameter stenosis of infarct-related lesion and other coronary lesions judged to cause luminal narrowing by >50%. Collateral grades will be prospectively recorded.

11 ENDPOINT DEFINITIONS

Death

Death will be defined as death from any cause at any time during the 90-day study period.

Myocardial Infarction

The appropriate definition of myocardial infarction depends upon the clinical scenario to which it is being applied. It should be noted that as CKMB is required for the infarct size assessment endpoint, this will be collected for the assessment of re-infarction. Troponin levels[17] will also be recorded but as recent trials have used CKMB to define re-infarction, especially in the first 18-24 hours, to provide consistency troponin-based re MI determinations will be considered exploratory, troponin levels will be used if CKMB levels are unavailable.

'Spontaneous' Re-Infarction:

- Re-infarction at <18 hours was defined as ischaemic chest pain lasting ≥30 minutes and recurrent ST-elevation of ≥1 mV in ≥2 contiguous leads, in patients with evidence of ≥1 mV of ST recovery.
- Re-infarction occurring at >18 hours required an elevation of CK-MB levels to > upper limit of reference (ULR) or new Q-waves of ≥30 ms in ≥2 contiguous leads distinct from the index STEMI or new LBBB.

When elevated troponin or CK-MB (or CK) levels are documented to be falling or have returned to <ULR, the diagnosis of re-infarction requires:

- a new elevation of troponin or CK-MB >ULR (or CK >ULR in the absence of MB determination) if the troponin or CK-MB (or CK) level has returned to <ULR,

OR

- a rise by >50% above the previous nadir level if the troponin or CK-MB (or CK) level has not returned to <ULR.

MI following PCI

- any CK-MB ≥3x ULR (or CK) within 24 hours after PCI which is also increased at least 50% over the most recent pre-PCI levels.

OR

- new, significant (≥0.03 s) Q waves in ≥2 contiguous ECG leads with CK-MB >ULR (or CK >2x ULR).

When the peak CK-MB (or CK) has not occurred prior to PCI: diagnosis of MI after PCI requires:

(a) recurrent chest pain ≥20 minutes

OR

new ST elevation in the setting of ST recovery as defined above

(b) the next CK-MB (CK) level measured at 8-12 hours after the event is elevated ≥50%; OR new, significant (≥0.03 s) Q waves in ≥2 contiguous ECG leads.

MI following Coronary Artery Bypass Grafting (CABG)

In patients undergoing CABG, the diagnosis of MI will require:

- any CK-MB ≥10x ULR (or CK ≥10x ULR in the absence of CKMB) within 24 hours of CABG and ≥50% over the prior to CABG levels;

OR

- any CK-MB ≥5x ULR (or CK ≥5x ULR in the absence of CKMB) within 24 hours of CABG and increased ≥50% over the most recent pre-CABG levels AND new, significant (≥0.03 s) Q waves in ≥2 contiguous ECG leads.

ACUITY Bleeding Classification

Major Bleeding

- Intracranial or intraocular
- Access site bleeding requiring intervention
- Reduction in Hb of ≥4.0 g/dL without an overt source of bleeding
- ≥3.0 g/dL with an overt source of bleeding
- Use of any blood product transfusion
- Haematoma ≥ 5cm in diameter,
- re-operation for bleeding

Minor Bleeding

Minor bleeding will be defined as all other bleeding. Data will be analysed according to the following criteria:

- Ecchymosis
- Epistaxis
- Gingival bleeding
- GI Bleed (greater than Guaiac positive)

- GU bleeding
- Haematemesis
- Haematoma <5cm puncture site
- Haemopericardium
- Haemoptysis
- Melaena
- Oozing blood at puncture site
- Otic bleeding
- Pericardial Haemorrhage
- Pharyngeal and other oral bleeding
- Prolonged bleeding at puncture site (>30minutes)
- Pulmonary Bleeding
- Other

Stroke *with documentation on imaging (e.g. CT or MRI) of*

- **Haemorrhagic**: a stroke haemorrhage in the cerebral parenchyma or a subdural or subarachnoid haemorrhage.
- **Ischemic**: documented history of stroke or cerebrovascular accident (CVA) resulting from an ischaemic event where the patient suffered a loss of neurological function with residual symptoms remaining for at least 24 hours after onset and which occurred before the current presentation/ admission.

12 SCREENING LOG

All patients who are considered for inclusion in the RAFT trial or who are not randomised due to Clinician decision will be recorded in a site screening log. Dependent on local ethical requirements for consent; anonymised and de-identified data may be pooled for a Registry.

13 STATISTICS

13.1 Statistical Design/Model

This study is powered, based on the primary safety endpoint of bleeding. Based on a transfusion rate of approximately 12% from the Liverpool Hospital (Sydney, Australia) rescue PCI experience of patients (with approximately 75% GPIIb/IIIa use) we hypothesised 15% transfusion rate in the UFH/LMWH and +/- a GPIIb/IIIa arm. Based on ACUITY[6] trial data, relating bleeding events and transfusions, it is hypothesised that a combined ACUITY (moderate and minor) bleeding rate of 30% will occur in the combined UHF/LMWH/+/- a GPIIb/IIIa inhibitor arm. Thus the hypothesised combined ACUITY bleeding rate in the bivalirudin arm is 18%.

A total sample size of 410 patients has 80% power ($p \leq 0.05$) of determining a 40% reduction in the ACUITY bleeding endpoint in the bivalirudin arm compared to the combined therapy arms. However, the study will employ an adaptive sample size re-estimation design with an interim analysis performed at 280 patients. The conditional probability of observing a 40% reduction in the primary endpoint based on the event rates observed at the interim analysis will be calculated and a sample size re-estimation undertaken to preserve the 80% power at the end of the study. Overall alpha for the study will be preserved using the Muller and Schafer Principle. The efficacy endpoint is the non-inferiority of the infarct size in the bivalirudin arm, compared to the standard therapy arm.

14 MONITORING

India

Site monitoring visits will be scheduled by the clinical monitor on a regular basis. During these visits, information recorded in the CRF will be verified against source documents for accuracy and completion. The clinical monitor will verify that the investigator follows the approved protocol or amendments (if any). Monitoring visits will be recorded in the Site Visit Log at the investigator's site, and at the end of the trial a copy of the completed Site Visit Log form will be returned to the Sponsor. At least 2 monitoring visits shall be conducted at all sites located in India. The investigator(s)/institution(s) will permit trial-related monitoring, audits, IRB/IEC review, and regulatory inspection(s), providing direct access to source data/documents.

Australia

Remote monitoring will continue in Australia with no on site monitoring.

15 ADVERSE DRUG REACTIONS REPORTING (SEE APPENDIX 1 FOR LIST OF CONDITIONS NOT TO BE REPORTED AS SAE'S)

DEFINITIONS

Adverse Event

An adverse event (AE) is defined as any untoward medical occurrence in a patient or clinical investigation subject administered a medicinal product and which does not necessarily have to have a causal relationship with this treatment. An AE can therefore be any unfavorable and unintended sign (including an abnormal laboratory finding), symptom or disease temporally associated with the use of a medicinal product, whether or not considered related to the medicinal product.

Planned hospital admissions and/or surgical operations for an illness or disease that existed before the drug was given or the subject was randomized in a clinical study are not to be considered AEs.

Serious Adverse Event

A serious adverse event (SAE) is any untoward medical occurrence that at any dose:

- results in death,
- is life-threatening, i.e., the subject was, in the opinion of the investigator, at immediate risk of death from the event as it occurred (It does not include an event that, had it occurred in a more severe form, might have caused death),
- results in persistent or significant disability/incapacity,
- requires in-subject hospitalization or prolongs hospitalization,
- is a congenital anomaly/birth defect, or
- is another medically significant event that, based upon appropriate medical judgment, may jeopardize the subject and may require medical or surgical intervention to prevent one of the outcomes listed above (e.g., allergic bronchospasm requiring intensive treatment in an emergency department or home, blood dyscrasias or convulsions that do not result in hospitalization, or the development of drug dependency or drug abuse).

Any AE fulfilling any one or more of these criteria must be reported as a SAE irrespective of the dose of the medication given and if the adverse event was a result of a drug interaction or misuse.

A distinction should be drawn between serious and severe AEs. Severity is an estimate or measure of the intensity of an AE, while the criteria for serious AEs are indications of adverse subject outcomes for regulatory reporting purposes. A severe AE need not necessarily be considered serious and a serious AE need not be considered severe. For example, nausea that persists for several hours may be considered severe nausea, but not an SAE. On the other hand, a urinary tract infection that may be considered minor could also be an SAE if it prolonged hospitalization.

Severity

Adverse events (AE) will be graded on a 3-point scale and reported as indicated on the case report form. The intensity of an AE is defined as follows:
1 = Mild: Discomfort noticed, but no disruption to daily activity.
2 = Moderate: Discomfort sufficient to reduce or affect normal daily activity.
3 = Severe: Inability to work or perform normal daily activity.

Study Drug Causality

The relationship of an AE to study treatment will be assessed with consideration to the following criteria:

- temporal relationship to the initiation of study medication
- response of the event to withdrawal of study medication
- AE profile of concomitant therapies
- clinical circumstances during which the AE occurred
- patient's clinical condition and medical history

Categorisation* of causality will be designated by the investigator as stated below:

1. Unrelated - this category applies to AEs that are clearly due to causes other than the study medication.
2. Unlikely related - this category applies to AEs for which there is no reasonable evidence or argument to suggest a causal relationship between the study medication and the AE.
3. Possibly related - this category applies to AEs for which there is reasonable evidence or argument to suggest a causal relationship between the AE and the study medication.
4. Definitely related - this category applies to AEs that are considered to be related to the study medication, with a high degree of certainty.

* For the purposes of regulatory reporting, categories '3' and '4' will be considered "related".

Procedure for Adverse Event Recording

All AEs (non-serious and serious) spontaneously reported by the subject and/or in response to an open question from study personnel or revealed by observation, physical examination or other diagnostic procedure must be recorded on the source documents and eCRF provided by the Investigator.

AEs that occur during the designated study period (30 days from consent) must be assessed and recorded on the source documents and CRF, regardless of causal relationship to the study drug.

The severity of an AE and the relationship to study drug will be assessed by the investigator. The investigator should ensure that any patient experiencing an AE receives appropriate medical support until the event resolves.

Procedure for Serious Adverse Event Reporting

In addition to entering each SAE on the appropriate page of the CRF, the investigator must complete a Serious Adverse Event Report (SAER) for each SAE regardless of causality by study medications. The SAER must be emailed to Adelaide Clinical Research (ACR) at RAFT@sahmri.com within 24 hours of the SAE being known ACR will contact the investigator, if necessary, to clarify any of the event information. The investigator should provide any follow-up information for the event to the ACR as soon as it becomes available. Additionally, if required by local regulations or procedures, the investigator should report these events to the Ethics Committee (EC).

This reporting requirement is applicable to SAEs that occur during the designated study period, 30 days from consent. If the investigator is notified of a serious event that occurs post-study that he or she wishes to report to Adelaide Clinical Research (e.g. an event suspected to be causally related to study drug), the event should be reported through the process described above.
All SAEs shall be reported to the applicable regulatory authority in the respective countries (Australia, New Zealand and India) as per local regulatory reporting requirements and timelines.

16 ETHICS

The study will be conducted in agreement with Declaration of Helsinki 2013, ICH E6 Guideline Good Clinical Practice (CPMP/ICH/135/95) and other guidelines applicable in respective participant countries.

17 REFERENCES

1. Wijeysundera HC, Vijayaraghavan R, Nallamothu BK, et al. Rescue angioplasty or repeat fibrinolysis after failed fibrinolytic therapy for ST-segment myocardial infarction: a meta-analysis of randomized trials. *J Am Coll Cardiol.* Jan 30 2007;49(4):422-430.

2. Gershlick AH, Stephens-Lloyd A, Hughes S, et al. Rescue angioplasty after failed thrombolytic therapy for acute myocardial infarction. *N Engl J Med.* Dec 29 2005;353(26):2758-2768.

3. Wijeysundera HC, Vijayaraghavan R, Nallamothu BK, et al. Rescue angioplasty or repeat fibrinolysis after failed fibrinolytic therapy for ST-segment myocardial infarction: a meta-analysis of randomized trials. *J Am Coll Cardiol.* Jan 30 2007;49(4):422-430.

4. Leung DY, French JK. End points in clinical trials: are they moving the goalposts? *Heart.* Jul 2006;92(7):870-872.

5. Lincoff AM, Kleiman NS, Kereiakes DJ, et al. Long-term efficacy of bivalirudin and provisional glycoprotein IIb/IIIa blockade vs heparin and planned glycoprotein IIb/IIIa blockade during percutaneous coronary revascularization: REPLACE-2 randomized trial. *Jama.* Aug 11 2004;292(6):696-703.

6. Stone GW, McLaurin BT, Cox DA, et al. Bivalirudin for patients with acute coronary syndromes. *N Engl J Med.* Nov 23 2006;355(21):2203-2216.

7. Stone GW, Witzenbichler B, Guagliumi G, et al. Bivalirudin during primary PCI in acute myocardial infarction. *N Engl J Med.* May 22 2008;358(21):2218-2230.

8. Indications for fibrinolytic therapy in suspected acute myocardial infarction: collaborative overview of early mortality and major morbidity results from all randomised trials of more than 1000 patients. Fibrinolytic Therapy Trialists' (FTT) Collaborative Group. *Lancet.* Feb 5 1994;343(8893):311-322.

9. Keeley EC, Boura JA, Grines CL. Primary angioplasty versus intravenous thrombolytic therapy for acute myocardial infarction: a quantitative review of 23 randomised trials. *Lancet.* Jan 4 2003;361(9351):13-20.

10. Keeley EC, Boura JA, Grines CL. Comparison of primary and facilitated percutaneous coronary interventions for ST-elevation myocardial infarction: quantitative review of randomised trials. *Lancet.* Feb 18 2006;367(9510):579-588.

11. Bonnefoy E, Steg PG, Boutitie F, et al. Comparison of primary angioplasty and pre-hospital fibrinolysis in acute myocardial infarction (CAPTIM) trial: a 5-year follow-up. *Eur Heart J.* Jul 2009;30(13):1598-1606.

12. Danchin N, Coste P, Ferrieres J, et al. Comparison of thrombolysis followed by broad use of percutaneous coronary intervention with primary percutaneous coronary intervention for ST-segment-elevation acute myocardial infarction: data from the french registry on acute ST-elevation myocardial infarction (FAST-MI). *Circulation.* Jul 15 2008;118(3):268-276.

13. Westerhout CM, Bonnefoy E, Welsh RC, Steg PG, Boutitie F, Armstrong PW. The influence of time from symptom onset and reperfusion strategy on 1-year survival in ST-elevation myocardial infarction: a pooled analysis of an early fibrinolytic strategy versus primary percutaneous coronary intervention from CAPTIM and WEST. *Am Heart J.* Feb 2011;161(2):283-290.

14. Tobing D, French J, Varigos J, Meehan A, Billah B, Krum H. Do patients with heart failure appropriately undergo invasive procedures post-myocardial infarction? Results from a prospective multicentre study. *Intern Med J.* Nov 2008;38(11):845-851.

15. Di Mario C, Dudek D, Piscione F, et al. Immediate angioplasty versus standard therapy with rescue angioplasty after thrombolysis in the Combined Abciximab REteplase Stent Study in Acute Myocardial Infarction (CARESS-in-AMI): an open, prospective, randomised, multicentre trial. *Lancet.* Feb 16 2008;371(9612):559-568.

16. Cantor WJ, Fitchett D, Borgundvaag B, et al. Routine early angioplasty after fibrinolysis for acute myocardial infarction. *N Engl J Med.* Jun 25 2009;360(26):2705-2718.

17. Antman EM, Hand M, Armstrong PW, et al. 2007 focused update of the ACC/AHA 2004 guidelines for the management of patients with ST-elevation myocardial infarction: a report of the American College of Cardiology/American Heart Association Task Force on Practice Guidelines. *J Am Coll Cardiol.* Jan 15 2008;51(2):210-247.

18. Van de Werf F, Bax J, Betriu A, et al. Management of acute myocardial infarction in patients presenting with persistent ST-segment elevation: the Task Force on the Management of ST-Segment Elevation Acute Myocardial Infarction of the European Society of Cardiology. *Eur Heart J.* Dec 2008;29(23):2909-2945.

19. Wijns W, Kolh P, Danchin N, et al. Guidelines on myocardial revascularization: The Task Force on Myocardial Revascularization of the European Society of Cardiology (ESC) and the European Association for Cardio-Thoracic Surgery (EACTS). *Eur Heart J.* Oct 2010;31(20):2501-2555.

20. Henry TD, Sharkey SW, Burke MN, et al. A regional system to provide timely access to percutaneous coronary intervention for ST-elevation myocardial infarction. *Circulation.* Aug 14 2007;116(7):721-728.

21. Ting HH, Rihal CS, Gersh BJ, et al. Regional systems of care to optimize timeliness of reperfusion therapy for ST-elevation myocardial infarction: the Mayo Clinic STEMI Protocol. *Circulation.* Aug 14 2007;116(7):729-736.

22. Jollis JG, Roettig ML, Aluko AO, et al. Implementation of a statewide system for coronary reperfusion for ST-segment elevation myocardial infarction. *Jama.* Nov 28 2007;298(20):2371-2380.

23. Edmond JJ, Juergens CP, French JK. The pharmaco-invasive approach to STEMI: when should fibrinolytic-treated patients go to the "cath lab"? *Heart.* Mar 2009;95(5):358-361.

24. Ellis SG, Da Silva ER, Spaulding CM, Nobuyoshi M, Weiner B, Talley JD. Review of immediate angioplasty after fibrinolytic therapy for acute myocardial infarction: insights from the RESCUE I, RESCUE II, and other contemporary clinical experiences. *Am Heart J.* Jun 2000;139(6):1046-1053.

25. Sutton AG, Campbell PG, Graham R, et al. A randomized trial of rescue angioplasty versus a conservative approach for failed fibrinolysis in ST-segment elevation myocardial infarction: the Middlesbrough Early Revascularization to Limit INfarction (MERLIN) trial. *J Am Coll Cardiol.* Jul 21 2004;44(2):287-296.

26. Andersen HR, Nielsen TT, Rasmussen K, et al. A comparison of coronary angioplasty with fibrinolytic therapy in acute myocardial infarction. *N Engl J Med.* Aug 21 2003;349(8):733-742.

27. Ganz W. Intracoronary thrombolysis in evolving myocardial infarction. *Ann Intern Med.* Oct 1981;95(4):500-502.

28. Rentrop P, Blanke H, Karsch KR, Kaiser H, Kostering H, Leitz K. Selective intracoronary thrombolysis in acute myocardial infarction and unstable angina pectoris. *Circulation.* Feb 1981;63(2):307-317.

29. Califf RM, O'Neil W, Stack RS, et al. Failure of simple clinical measurements to predict perfusion status after intravenous thrombolysis. *Ann Intern Med.* May 1988;108(5):658-662.

30. Shah PK, Cercek B, Lew AS, Ganz W. Angiographic validation of bedside markers of reperfusion. *J Am Coll Cardiol.* Jan 1993;21(1):55-61.

31. de Lemos JA, Antman EM, Giugliano RP, et al. ST-segment resolution and infarct-related artery patency and flow after thrombolytic therapy. Thrombolysis in Myocardial Infarction (TIMI) 14 investigators. *Am J Cardiol.* Feb 1 2000;85(3):299-304.

32. Zeymer U, Schroder R, Tebbe U, Molhoek GP, Wegscheider K, Neuhaus KL. Non-invasive detection of early infarct vessel patency by resolution of ST-segment elevation in patients with thrombolysis for acute myocardial infarction; results of the angiographic substudy of the Hirudin for Improvement of Thrombolysis (HIT)-4 trial. *Eur Heart J.* May 2001;22(9):769-775.

33. French JK, Andrews J, Manda SO, Stewart RA, McTigue JJ, White HD. Early ST-segment recovery, infarct artery blood flow, and long-term outcome after acute myocardial infarction. *Am Heart J.* Feb 2002;143(2):265-271.

34. Andrews J, Straznicky IT, French JK, et al. ST-Segment recovery adds to the assessment of TIMI 2 and 3 flow in predicting infarct wall motion after thrombolytic therapy. *Circulation.* May 9 2000;101(18):2138-2143.
35. Roe MT, Ohman EM, Maas AC, et al. Shifting the open-artery hypothesis downstream: the quest for optimal reperfusion. *J Am Coll Cardiol.* Jan 2001;37(1):9-18.
36. Late Assessment of Thrombolytic Efficacy (LATE) study with alteplase 6-24 hours after onset of acute myocardial infarction. *Lancet.* Sep 25 1993;342(8874):759-766.
37. Hochman JS, Lamas GA, Buller CE, et al. Coronary intervention for persistent occlusion after myocardial infarction. *N Engl J Med.* Dec 7 2006;355(23):2395-2407.
38. Bittl JA, Strony J, Brinker JA, et al. Treatment with bivalirudin (Hirulog) as compared with heparin during coronary angioplasty for unstable or postinfarction angina. Hirulog Angioplasty Study Investigators. *N Engl J Med.* Sep 21 1995;333(12):764-769.

APPENDIX 1

Serious Adverse Events

NOTE: The following are captured as study endpoints and are not required to be recorded as SAE's:

1. Death
2. 'Spontaneous' Re-infarction
3. MI following PCI
4. MI following CABG
5. ACUITY Major and Minor Bleeding
6. Stroke

The following are some of the expected clinical events that may occur, these are to be reported as adverse events only and not SAE's:

1. CABG-elective or non-urgent (based on coronary anatomy)
2. Recurrent ischaemia
3. Atrial fibrillation
4. Sustained ventricular tachycardia
5. Ventricular fibrillation
6. Electrical mechanical dissociation
7. Cardiac tamponade/ Ventricular rupture
8. Acute ventricular septal defect
9. Acute mitral valve regurgitation
10. Acute heart failure
11. Asystole
12. Pericarditis
13. Cardiogenic shock
14. Symptomatic hypotension
15. Renal failure
16. Significant infections e.g. pneumonia

Any other adverse events that meet the criteria for an SAE should be reported for example:

1. CABG as a result of a complication of a PCI
